# Targeting gut dysbiosis against inflammation and impaired autophagy in Duchenne muscular dystrophy

**DOI:** 10.15252/emmm.202216225

**Published:** 2023-01-03

**Authors:** Hilal Kalkan, Ester Pagano, Debora Paris, Elisabetta Panza, Mariarosaria Cuozzo, Claudia Moriello, Fabiana Piscitelli, Armita Abolghasemi, Elisabetta Gazzerro, Cristoforo Silvestri, Raffaele Capasso, Andrea Motta, Roberto Russo, Vincenzo Di Marzo, Fabio Arturo Iannotti

**Affiliations:** ^1^ Endocannabinoid Research Group, Institute of Biomolecular Chemistry (ICB) National Research Council (CNR) Pozzuoli Italy; ^2^ Department of Pharmacy University Federico II of Naples Italy; ^3^ Institut Universitaire de Cardiologie et de Pneumologie de Québec and Institut Sur la Nutrition et Les Aliments Fonctionnels, Centre NUTRISS Université Laval Quebec City QC Canada; ^4^ Unit of Muscle Research Experimental and Clinical Research Center Charité Universitätsmedizin and Max Delbrück Research Center Berlin Germany; ^5^ Department of Agricultural Sciences University of Naples Federico II Portici Italy

**Keywords:** autophagy, duchenne muscular dystrophy, endocannabinoid system, gut microbiota, short‐chain fatty acids, Microbiology, Virology & Host Pathogen Interaction, Musculoskeletal System

## Abstract

Nothing is known about the potential implication of gut microbiota in skeletal muscle disorders. Here, we provide evidence that fecal microbiota composition along with circulating levels of short‐chain fatty acids (SCFAs) and related metabolites are altered in the mdx mouse model of Duchenne muscular dystrophy (DMD) compared with healthy controls. Supplementation with sodium butyrate (NaB) in mdx mice rescued muscle strength and autophagy, and prevented inflammation associated with excessive endocannabinoid signaling at CB1 receptors to the same extent as deflazacort (DFZ), the standard palliative care for DMD. In LPS‐stimulated C2C12 myoblasts, NaB reduces inflammation, promotes autophagy, and prevents dysregulation of microRNAs targeting the endocannabinoid CB1 receptor gene, in a manner depending on the activation of GPR109A and PPARγ receptors. In sum, we propose a novel disease‐modifying approach in DMD that may have benefits also in other muscular dystrophies.

## Graphical abstract

Changes in gut microbiota composition are found to be associated with an excess of endocannabinoid system activity and increased expression of cannabinoid CB1 receptors, suggesting a novel pathological mechanism implicated in the severity and progression of Duchenne muscular dystrophy.

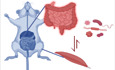

## Introduction

Myopathies is a general term that refers to a large group of rare skeletal muscle disorders many of which are associated with poor prognosis. Among them, Duchenne muscular dystrophy (DMD) is the most frequent and detrimental form, affecting approximately 1 in 3,500 male births worldwide (Crisafulli *et al*, 2020). In most cases, DMD is caused by deletions of one or more exons within the gene encoding for dystrophin, a rod‐shaped protein that physically interacts with other specialized proteins to form the dystrophin‐associated glycoprotein complex (DAPC), playing a crucial structural and signaling role both in cardiac and skeletal muscles (Cirak *et al*, 2012). Therefore, the alteration of dystrophin expression and function leads to the collapse of muscle structure and irreversible tissue degeneration, a condition that is further aggravated by ensuing persistent inflammation, impairment of autophagy, fibrosis, and tissue necrosis (Sandri *et al*, 2013; De Palma *et al*, 2014). Unfortunately, a cure for DMD is still not available, although experimental therapies have made important advances over the years (Sheikh & Yokota, 2020). For this reason, corticosteroids mainly including prednisolone (PRED) and deflazacort (DFZ) remain the mainstay of palliative care. Both agents were shown to produce beneficial effects on the preservation of functional abilities in several mouse models of DMD and randomized controlled trials (Bushby *et al*, 2010; Griggs *et al*, 2016). However, there is uncertainty regarding the long‐term benefits and safety of these treatments. Results from randomized controlled studies revealed that side effects including sudden weight gain, confusion, depression, growth‐related complication, and cataracts may be caused by both DFZ and PRED (Matthews *et al*, 2016; Biggar *et al*, 2022).

The central role of the gut microbiota in human health and disease is a fascinating and rapidly expanding field of research. In this regard, over the last decades, much has been learned about the association between the gut microbiota and skeletal muscle mass as well as metabolic and contractile properties (Bäckhed *et al*, 2007; Grosicki *et al*, 2018; Lahiri *et al*, 2019). However, the molecular mechanisms linking intestinal microorganisms to skeletal muscle remain largely unknown. In this context, recent studies have demonstrated the role of major classes of metabolites produced by, or related to the gut microbiota, such as short‐chain fatty acids (SCFAs), i.e., acetate, propionate and butyrate, and ketone bodies (KBs; acetoacetate acid, β‐hydroxybutyric acid, and acetone), in contributing to skeletal muscle mass, glucose and lipid metabolism, and physical performance (Frampton *et al*, 2020). One of the proposed mechanisms through which SCFAs and KBs provide various health benefits on host energy metabolism is through intracellular signaling dependent on the activation of GPR41, GPR43, GPR109A, and/or peroxisome proliferator‐activated receptor‐gamma (PPARγ) receptors (Schwab *et al*, 2006; Layden *et al*, 2013).

Additionally, more recent evidence points to the endocannabinoid system as a key regulator in the communication between the gut microbiota and the host (Cani *et al*, 2016; de Vos *et al*, 2022).

The endocannabinoid system (ECS) refers to a complex lipid cell‐signaling system playing an important role in human health and disease. Although the number of molecules linked to the ECS is constantly expanding, the major players remain the two lipid mediators anandamide (AEA) and 2‐arachidonoylglycerol (2‐AG), which primarily activate two G‐coupled receptors, differently distributed in the body, named cannabinoid receptor of type 1 (CB1) and type 2 (CB2) (Iannotti *et al*, 2016). Despite the promising use of endocannabinoids or plant cannabinoids as complementary and/or alternative medicines (Di Marzo, 2018), to date, their potential use in skeletal muscle disorders is still largely unexplored. Recently, we demonstrated that (i) in both murine and human skeletal muscle cell precursors (myoblasts), 2‐AG promotes proliferation and inhibits differentiation to mature muscle cells (myotubes); (ii) 2‐AG negatively controls skeletal muscle formation *in vivo*; (iii) the ECS is overactive in both murine and human skeletal muscles affected by DMD; and (iv) the pharmacological inhibition of the endocannabinoid CB1 receptor promotes differentiation of both satellite and myoblast cells into mature myotubes, prevents locomotor impairment in dystrophic mice, and reduces muscle inflammation (Iannotti *et al*, 2014, 2018). However, whether the ECS could represent an additional signaling mechanism through which the gut microbiota interacts with the skeletal muscle remains to date completely unexplored. Based on this background, in this study, by using a multidisciplinary approach, we aimed at exploring whether changes in the gut microbiota diversity and its consequent dysfunctional interplay with the ECS could represent a novel potential molecular mechanism to be targeted in DMD.

## Results

### Fecal microbiota composition is significantly altered in mdx mice

In this study, the microbiota composition was analyzed in fecal samples collected from control (wild‐type—wt) and dystrophic (mdx) mice of 16 weeks of age randomly separated into the following experimental groups: (i) wt mice receiving vehicle (DMSO); (ii) wt mice receiving DFZ; (iii) mdx mice receiving vehicle; and (iv) mdx mice receiving DFZ. DMSO or DFZ (1.2 mg/kg) was administered once daily by oral gavage for 3 weeks.

**Figure 1:**
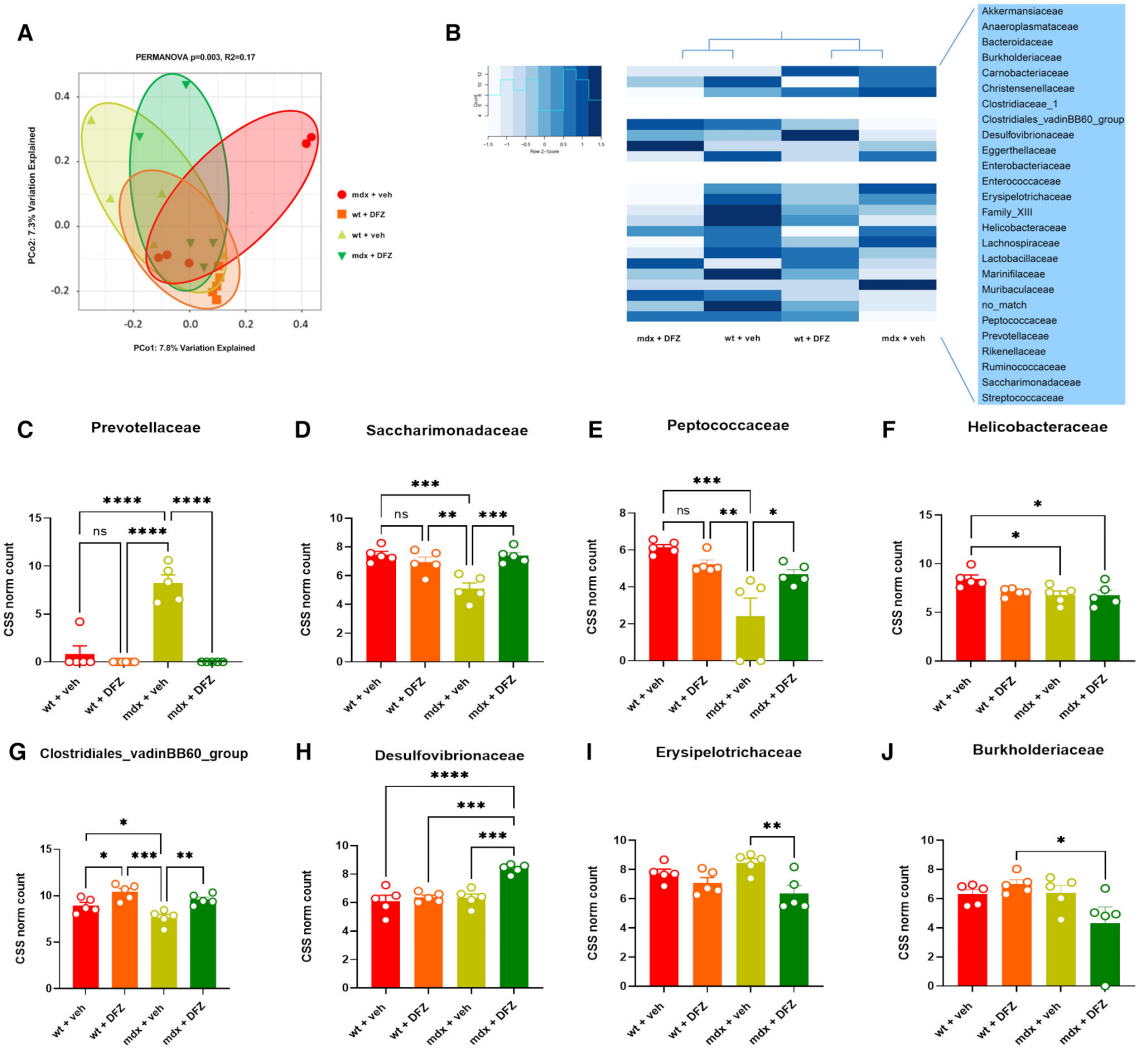
Microbiota analysis in fecal samples of control and mdx mice APrincipal coordinate analysis (PCoA) displays differences in gut microbiota composition diversity among the four animal groups. Bray–Curtis dissimilarity indexes were used to estimate B‐diversity. PERMANOVA *P* values (Adonis R function) are displayed above PCoA.BHeat map and hierarchical clustering of family composition using cumulative sum scaled (CSS) normalized bacterial counts.C–JBar Chart with data points showing the abundance of indicated bacterial families in the indicated group of mice. Data are expressed as CSS‐normalized bacterial counts. Data Information: Each bar is the mean ± S.E.M. from 5 independent biological samples. ****P ≤ 0.0001; ***P ≤ 0.0003; **P ≤ 0.003; *P ≤ 0.05 vs. the indicated experimental group calculated using ANOVA.

As shown in Fig 1A, principal coordinate analysis (PCoA) revealed a significant (PERMANOVA analysis; *P* < 0.003) clustering dissimilarity of fecal microbiota composition among the four groups of mice. Clustering analysis of bacterial families detected and changing between wt and mdx mice treated without or with DFZ revealed that mdx mice treated with DFZ clustered with wt mice (Fig 1B). *Prevotellaceae*, *Saccharimonadaceae*, *Peptococcaceae*, *Helicobacteriaceae*, and *Clostridiales_vadinBB60* were the bacterial families showing the most prominent alteration between wt and mdx mice, with *Prevotellaceae* being increased in the latter, while the other families were decreased (Fig 1C–G). Alterations in *Prevotellaceae*, *Saccharimonadaceae*, *Peptococcaceae*, and *Clostridiales_vadinBB60* levels were reversed by DFZ (Fig 1C and G). However, in mdx mice, the treatment with DFZ also significantly increased the relative abundance of *Desulfovibrionaceae* (Fig 1H) and reduced that of *Erysipelotrichaceae* and *Burkholdariaceae* (Fig 1I and J). Finally, we detected no significant differences in the microbiota composition between wt mice receiving DMSO or DFZ, with the only exception of *Clostridiales_vadinBB60* levels (Fig 1G).

### Plasma levels of SCFAs and KBs are dysregulated in mdx mice

**Figure 2:**
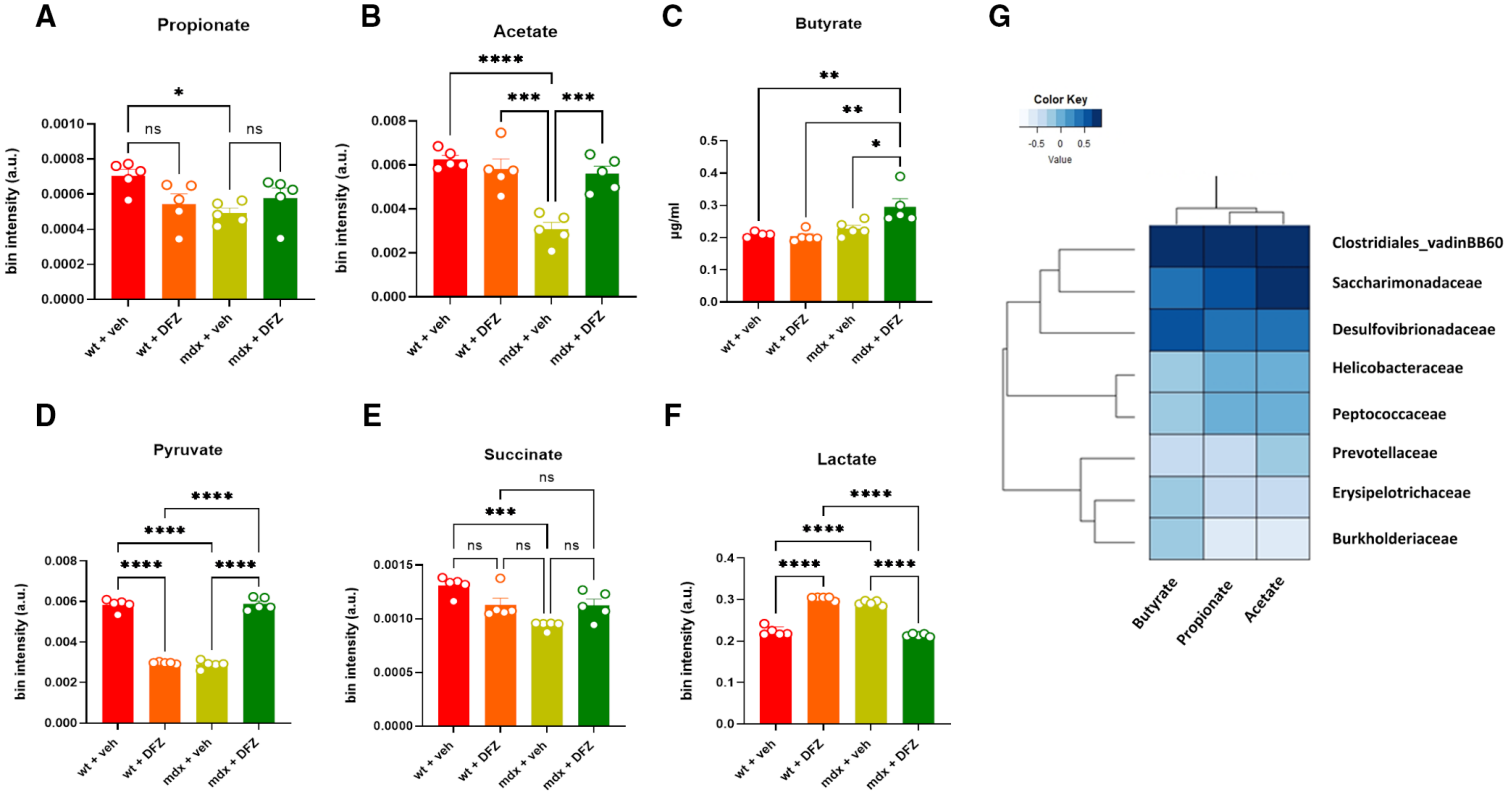
Analysis of SCFAs and their precursors in the blood plasma of wt and mdx treated with DFZ or not A–CBar chart with individual points showing the levels of propionate, acetate and butyrate in wt and mdx mice receiving DFZ or vehicle, measured by NMR or GC/MS. Data are expressed as μg/ml or bin intensity (arbitrary unit—a.u.)D–FBar chart with individual points showing the levels of pyruvate, succinate, and lactate in wt and mdx mice receiving vehicle or DFZ, measured by NMR.GCorrelation map based on Pearson correlation coefficients between butyrate, propionate, and acetate, and bacterial families changing in mdx mice. Rows and columns are rearranged according to the WARD‐based correlation matrix‐based hierarchical clustering (CMBHC). Data Information: Each bar is the mean ± S.E.M. from 5 independent biological samples. ****P ≤ 0.0001; ***P ≤ 0.0003; **P ≤ 0.003; *P ≤ 0.05 vs. the indicated experimental group calculated using ANOVA.

**Figure EV1:**
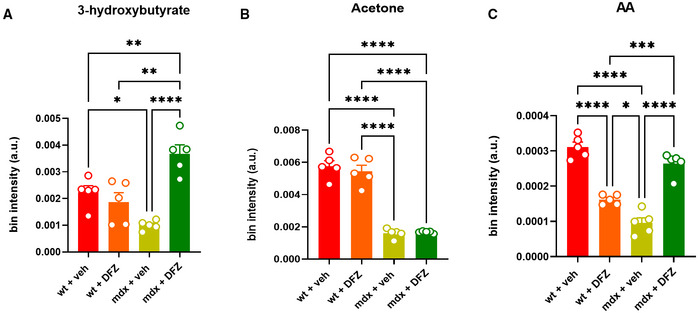
Measurement of KBs in plasma samples of wt and mdx mice treated with or without DFZ A–C Bar chart with individual points showing the levels of the indicated metabolites detected in the plasma of wt and mdx mice treated ± DFZ. Data are expressed as bin intensity (a. u., arbitrary unit). Data Information: Each bar is the mean ± S.E.M. of 5 independent biological determinations. ****P ≤ 0.0001; ***P ≤ 0.0003; **P < 0.01; *P ≤ 0.05 vs. the indicated experimental group calculated using ANOVA.

**Figure EV2:**
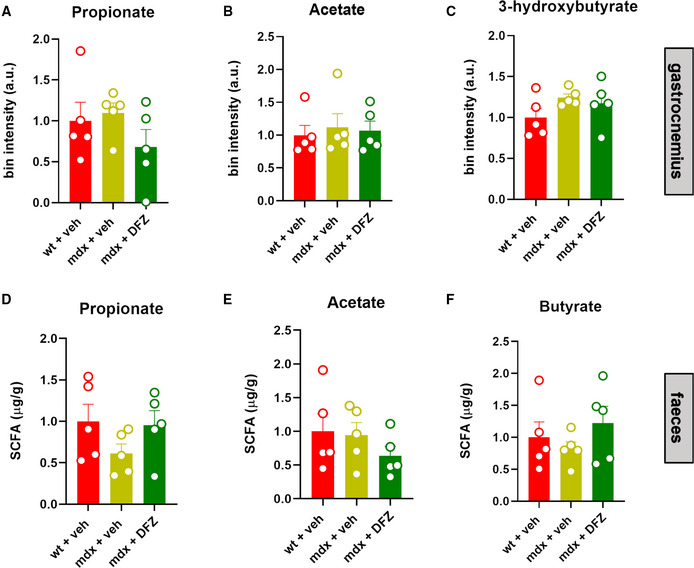
Measurement of SCFAs and KBs in the gastrocnemius and fecal samples of wt and mdx mice treated with or without NaB or DFZ A–FBar chart with individual points showing the levels of the indicated metabolites detected in the gastrocnemius and/or fecal samples of wt and mdx mice treated ± DFZ. Data are expressed as bin intensity (a. u., arbitrary unit). Data Information: Each bar is the mean ± S.E.M. from 5 independent biological samples.

To understand whether the changes in fecal microbiota found in mdx mice resulted in changes in the levels of metabolites produced or associated with intestinal microbiota, we quantified the concentrations of SCFAs, KBs, and related molecules in blood plasma, skeletal muscles, and feces samples of mice subjected to our experimental conditions. Using GC/MS and NMR analysis, we found that plasma levels of propionate and acetate, but not butyrate (the only one not detectable by NMR), were significantly reduced in mdx vs. control mice (Fig 2A–C). The treatment with DFZ of mdx mice prevented changes in propionate and acetate levels (Fig 2A and B) and remarkably increased butyrate levels in mdx mice (Fig 2C). Additionally, using NMR, we found that plasma levels of pyruvate, succinate, and lactate, which are key precursors in the biosynthetic pathway of SCFAs from carbohydrates (Koh *et al*, 2016), were dysregulated in dystrophic mice (Fig 2D–F). The treatment with DFZ, also in this latter case, prevented these alterations while inducing a significant reduction of pyruvate, whereas lactate was increased in control mice (Fig 2D–F). The matrix shows the potential correlation between SCFAs and bacterial families changing in mdx mice (Fig 2G). Similar to SCFAs, plasma levels of KBs including acetone, acetoacetic acid (AA), and 3‐hydroxybutyric acid were reduced in mdx mice. Following the treatment with DFZ, the levels of 3‐hydroxybutyric and AA were restored to the control condition (Fig EV1). By contrast, no statistically significant differences in either SCFAs or KB levels were found in gastrocnemius and fecal samples between control and mdx mice (Fig EV2).

These data, together with those described in the previous section, suggest that functional alterations of the gut microbiota are present in mdx mice as compared to wt mice and that many of such alterations are reversed following 3 weeks treatment with DFZ. Importantly, in dystrophic mice, DFZ elevated levels of butyrate, which was previously described to exert beneficial anti‐inflammatory and immunomodulatory activities (Prokopidis *et al*, 2021).

### Locomotor activity, muscle autophagy deficits, and inflammation in mdx mice are ameliorated after supplementation with sodium butyrate

**Figure 3:**
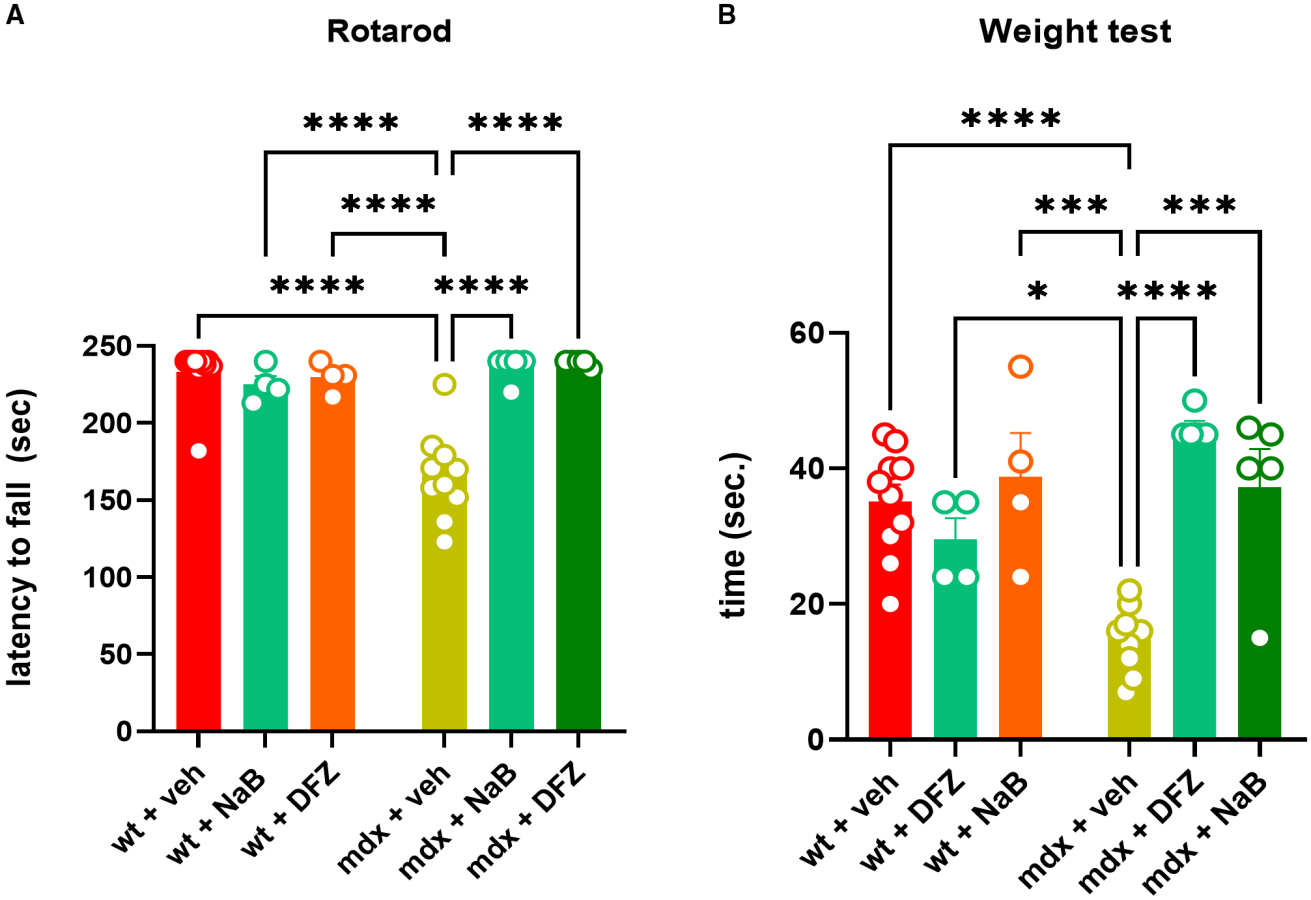
Measurement of locomotor activity A, BMuscle coordination and strength were measured in 19‐week‐old control and mdx mice treated with vehicle (DMSO), NaB (100 mg/kg/daily), or DFZ (1.2 mg/kg/daily) for 3 weeks using the rotarod and weight test. Bar charts show the latency to fall or drop the weight of wt and dystrophic mice. Data Information: Each bar is the mean ± S.E.M. from 5 or more independent biological determinations. ****P ≤ 0.0001; ***P ≤ 0.0003; *P ≤ 0.05 vs. the indicated experimental group calculated using ANOVA.

**Figure 4:**
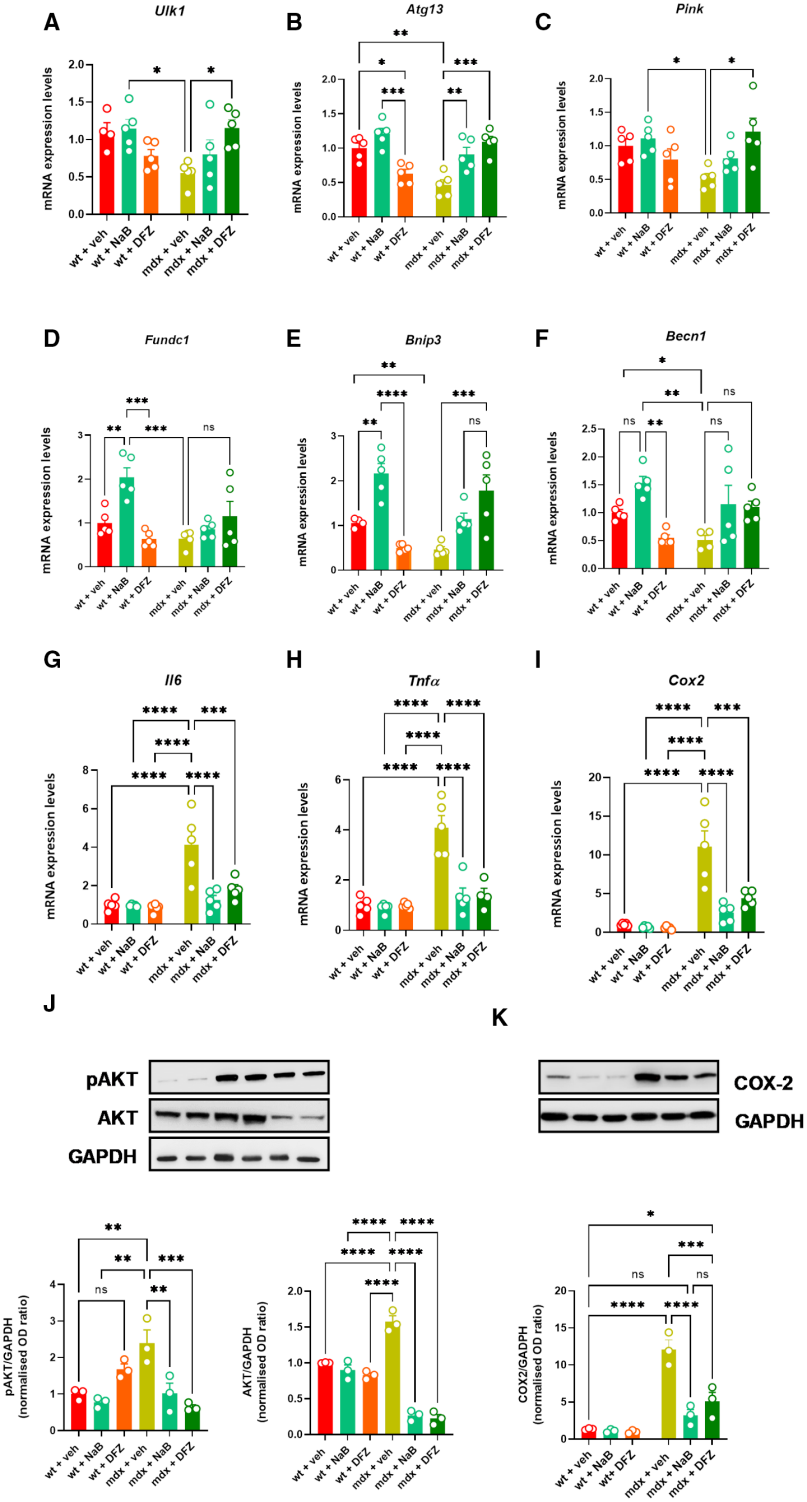
Expression of autophagy and inflammatory genes in wt and mdx mice treated with NaB or DFZ A–IBar chart with individual points showing the mRNA expression levels of the indicated genes measured in the gastrocnemius of control and mdx mice treated with or without NaB and DFZ.J, KRepresentative blotting and bar chart with individual points showing the expression and/or phosphorylation of pAKT/AKT and COX2 in the gastrocnemius of the indicated six groups of mice. Data Information: Each bar is the mean ± S.E.M. from 5 independent biological replicates. ****P ≤ 0.0001; ***P ≤ 0.0003; **P ≤ 0.005; *P ≤ 0.05 vs. the indicated experimental group calculated using ANOVA.

In order to understand whether the perturbation of the gut microbiota (known as dysbiosis) is associated with muscle function impairment in mdx mice through the ensuing deficit of SCFAs production, we next measured muscle coordination and strength in wt and mdx mice receiving a daily oral supplementation of sodium butyrate (NaB; 100 mg/kg) or DFZ (1.2 mg/kg) for 3 weeks using a rotarod and weight test. In agreement with our previous findings (Iannotti *et al*, 2018), we found that 19‐week‐old mdx mice showed marked impairment of muscle coordination and strength compared with their age‐matched controls (Fig 3A and B). Remarkably, the treatment with NaB prevented the loss of locomotor function in mdx mice, to an extent comparable to that of DFZ, while both NaB and DFZ had no effect in control mice (Fig 3A and B). Transcriptomic analysis performed on RNA extracted from dissected gastrocnemius revealed that NaB, to the same extent as DFZ, was able to restore the defective expression of key genes regulating autophagy and/or mitophagy (*Ulk1*, *Atg13*, *Pink1*, *Becn1*, *Fundc1*, and *Bnip)* in mdx mice (Fig 4A–F) and concomitantly prevented the up‐regulation of pro‐inflammatory genes such as interleukin 6 (*Il6*), tumor necrosis factor‐α (*Tnfα*), and cyclooxygenase‐2 (*Cox2*) (Fig 4G–I). Additionally, using western blot analysis, we found that NaB, similar to DFZ, prevented the increased phosphorylation (hence activation) of AKT, a negative regulator of autophagy (De Palma *et al*, 2014; Fig 4J), and concomitantly reduced the expression of COX2 (Fig 4K), which is considered a key therapeutic target to counteract inflammation in DMD (Péladeau *et al*, 2018). These data suggest that the dysfunctional gut microbiota is implicated in the pathogenesis of DMD in mdx mice. In the same vein, NaB supplementation prevented some of the features of muscular dystrophy in these mice, i.e., impaired locomotion and muscle autophagy and enhanced muscle inflammation.

### Endocannabinoid system overactivity in blood and skeletal muscle of mdx mice is reversed by NaB or DFZ

**Figure 5:**
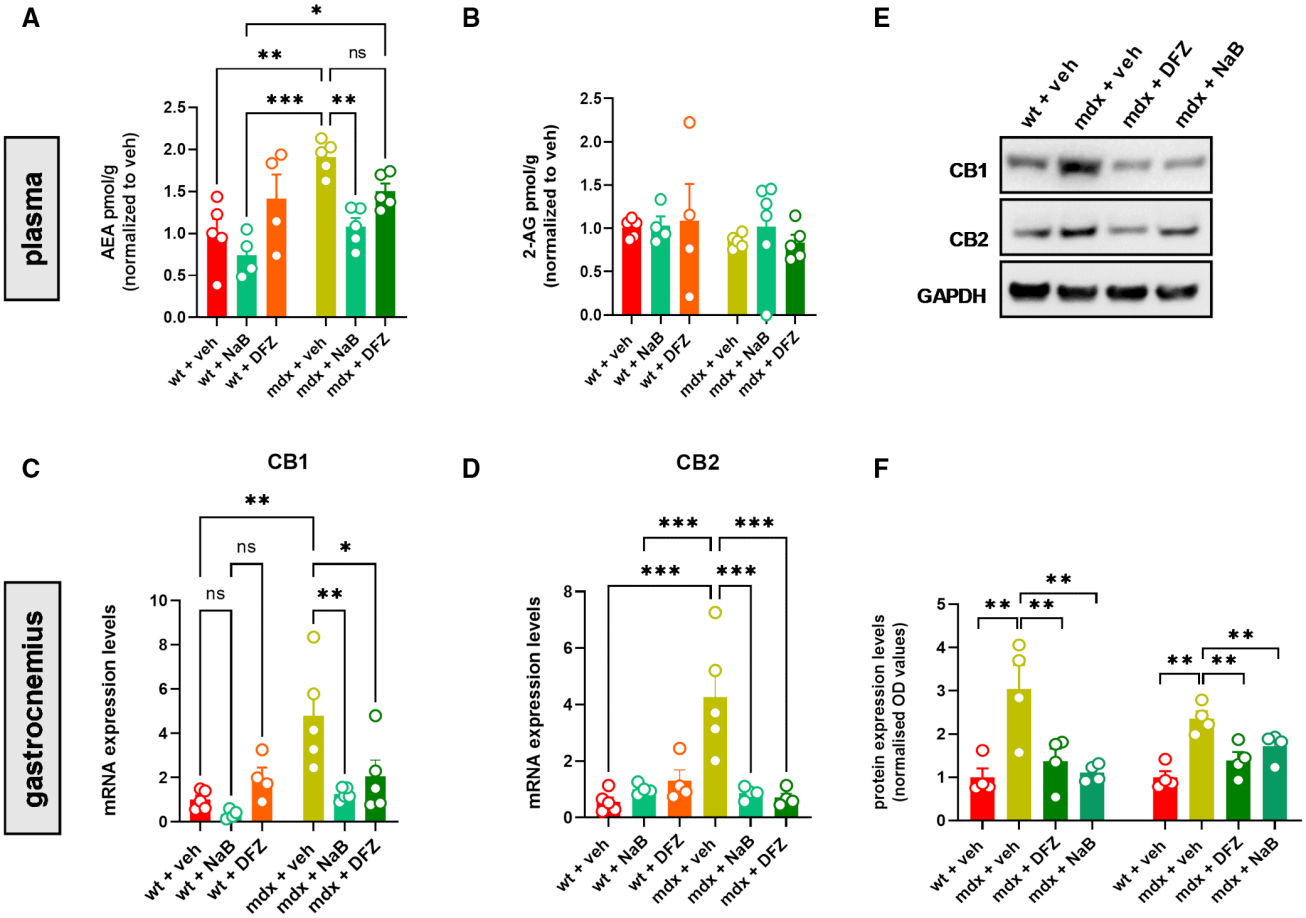
Measurement of endocannabinoid system activity in the plasma and skeletal muscle of wt and mdx mice treated with NaB and DFZ A, BLevels of AEA and 2‐AG in plasma samples of wt and mdx mice expressed as pmol/mg of wet tissue weight.C, DBar charts with individual points showing the mRNA expression levels of CB1 and CB2 measured in the gastrocnemius of the indicated groups of mice.ERepresentative blots showing the expression levels of CB1 and CB2 proteins in the gastrocnemius of the indicated groups of mice.FQuantification of CB1 and CB2 proteins to the housekeeping protein GAPDH. Data Information: Each bar is the mean ± S.E.M. of 4–5 independent biological samples. ***P ≤ 0.0003; **P ≤ 0.003; *P ≤ 0.05 vs. the indicated experimental group calculated using ANOVA.

Next, we measured the levels of the two major endocannabinoids AEA and 2‐AG in the plasma of mice subjected to our experimental conditions. Liquid chromatography–mass spectrometry (LC–MS) analysis revealed that the levels of AEA, but not 2‐AG, were significantly increased in 19‐week‐old mdx mice compared with the control group (Fig 5A and B). Changes in AEA levels were associated with a significant up‐regulation of CB1 and CB2 mRNA and protein expression in the gastrocnemius of dystrophic mice (Fig 5C–F). Notably, in mdx mice, the dysregulated levels of AEA as well as CB1 and CB2 genes were prevented by NaB and DFZ (Fig 5A–F). In control mice, treatment with NaB or DFZ did not significantly change the levels of AEA or 2‐AG, nor the expression of CB1 and CB2 genes (Fig 5A–D).

### Pharmacological blockade of CB1 rescues autophagy in skeletal muscles of mdx mouse

**Figure 6:**
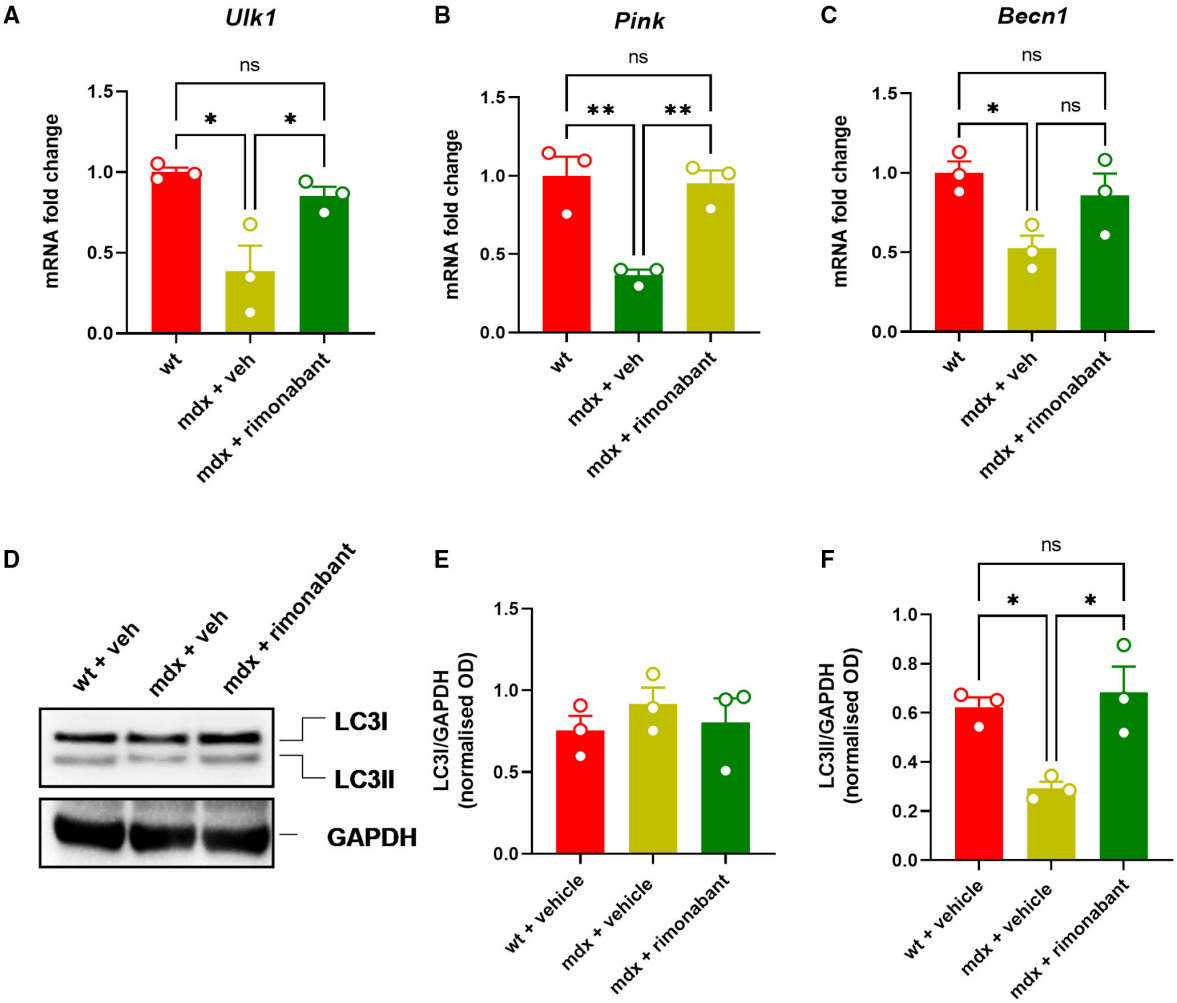
Effect of rimonabant on autophagy in mdx mice A–CBar charts with individual points showing the mRNA expression levels of *Ulk*, *Pink*, and *Becn1* measured in control and mdx mice treated with rimonabant (0.5 mg/kg).DRepresentative blots showing the expression levels of LC3I and LC3II proteins in the gastrocnemius of the indicated groups of mice.E, FQuantification of LC3I and LC3II proteins to the housekeeping protein GAPDH. Data Information: Each bar is the mean ± S.E.M. from 3 independent biological samples. **P ≤ 0.005 *P ≤ 0.05 vs. the indicated experimental group calculated using ANOVA.

**Figure EV3:**
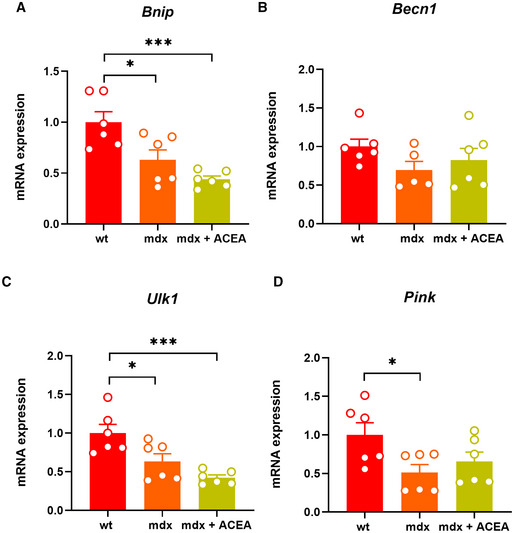
Effect of ACEA on the expression of autophagy‐related genes in mdx mice A–DBar charts with individual points showing the mRNA expression levels of *Bnip*, *Becn1*, *Ulk1*, and *Pink* measured in control and mdx mice treated with ACEA 2.5 mg/kg. Data Information: Each bar is the mean ± S.E.M. from 6 independent biological samples. ***P ≤ 0.0003; *P ≤ 0.05 vs. the indicated experimental group calculated using ANOVA.

Our previous findings revealed that the ECS is overactive in mdx mice, predominantly via CB1 receptors, leading to a concurrent increase in the inflammatory burden and reduction in myotube formation from satellite and myoblast cells (Iannotti *et al*, 2018). Here, we evaluated the effect of CB1 on skeletal muscle autophagy. Intriguingly, the reduced expression of *Ulk1*, *Pink*, and *Becn1* genes observed in skeletal muscles (gastrocnemius) of dystrophic mice was prevented by treatment with rimonabant (0.5 mg/kg), a selective CB1 receptor antagonist (Fig 6A–C). In addition, using western blot, we found that in mdx mice, rimonabant restored the expression of LC3II, which is physiologically converted from LC3I to initiate the formation and lengthening of the autophagosome (Runwal *et al*, 2019) (Fig 6D–F). Conversely, in mdx mice treated with ACEA (2.5 mg/kg), a selective CB1 agonist (Hillard *et al*, 1999), the inactivation of autophagy genes tended to aggravate (Fig EV3). These findings, together with those described in the previous section, suggest that enhanced endocannabinoid signaling at CB1 receptors may act as an intermediate of the effects of the dysfunctional gut microbiota on inflammation and impaired locomotor activity and autophagy in mdx mice.

### Butyrate protects skeletal muscle cells from LPS‐induced inflammation, promotes autophagy, and prevents endocannabinoid overactivity via multiple receptors

Next, we went on to elucidate the molecular mechanisms through which, upstream of endocannabinoid signaling at CB1 receptors, NaB exerts anti‐inflammatory and pro‐autophagic effects in skeletal muscle tissues. To this purpose, we employed murine C2C12 myoblasts and myotubes to measure the expression of potential molecular targets of SCFAs. There is evidence that some members of the large family of G protein‐coupled receptors (GPCRs) including GPR41 and GPR43 have an affinity for propionate, butyrate, and acetate, while GPR109A and peroxisome proliferator‐activated receptor‐gamma (PPARγ) show a selective affinity only for butyrate (Brown *et al*, 2003; Le Poul *et al*, 2003; Sun *et al*, 2017; Kumar *et al*, 2020). Therefore, using qPCR, we found that among GPCRs, GPR109A was the only gene expressed in both C2C12 myoblasts and myotubes (Table EV1). In agreement with others, we also found that PPARγ is expressed in myoblasts more than in myotubes (Table EV1) (Singh *et al*, 2007). Importantly, among SCFA targets, the expression of GPR109A and PPARγ was also predominant in the skeletal muscle (gastrocnemius) of control and dystrophic mice (Table EV1).

**Figure 7:**
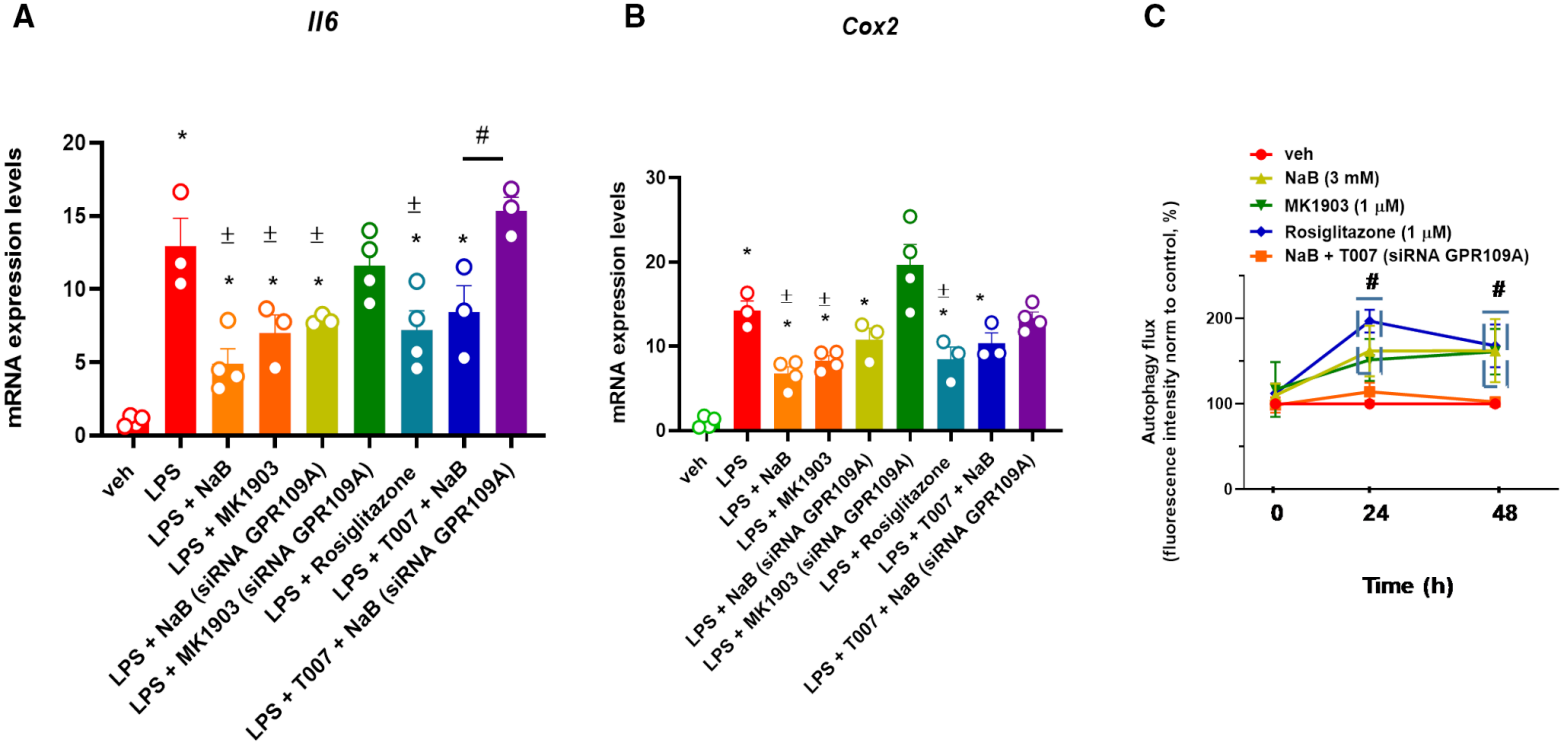
Effect of NaB on LPS‐stimulated C2C12 cells A, BBar chart with individual points showing the mRNA expression levels of *Il6* and *Cox2* in control (vehicle, DMSO) and/or GPR109A‐silenced C2C12 myoblasts exposed to LPS (1 μg/ml) in the presence or absence of either MK1903 (1 μM), rosiglitazone (1 μM), or T007 (1 μM).CTime‐dependent effect of NaB (3 mM), MK1903 (1 μM), and rosiglitazone (1 μM) on autophagosome formation measured in C2C12 myoblasts. Data are expressed as fluorescence intensity normalized to controls (%). Data Information: Each bar is the mean ± S.E.M. of at least 3 independent replicates. *P ≤ 0.05 vs. the veh group. ^±^ *P* ≤ 0.05 vs. the LPS group; ^#^ *P* ≤ 0.05 vs. the other experimental groups (A) or the veh group (B) calculated using ANOVA.

Subsequently, C2C12 myoblasts were stimulated with bacterial lipopolysaccharide (LPS) to mimic the inflammatory microenvironment, which prevails in DMD (Boursereau *et al*, 2018). Cytokines including IL‐6, IL‐1, and TNFα, as well as the enzyme COX2, are known to be increased during inflammation in DMD (Cruz‐Guzmán Odel *et al*, 2015). Therefore, following published procedures (Park *et al*, 2021), we observed that in C2C12 myoblasts stimulated with LPS (1 μg/ml for 3 h), the mRNA expression of *Il6* and *Cox2* was robustly increased (~ 15 fold). However, when C2C12 cells were pretreated with NaB (3 mM) or MK1903 (1 μM, a selective GPR109A agonist) for 30 min before the stimulation with LPS, the up‐regulation of *Il6* and *Cox2* was significantly prevented (Fig 7A and B). Additionally, in myoblasts silenced for GPR109A, the protective effect of NaB was partially abolished (Fig 7A and B). Rosiglitazone (1 μM), a selective PPARγ agonist, similar to NaB and MK1903 prevented the LPS‐induced up‐regulation of both *Il6* and *Cox2* (Fig 7A and B). Notably, the effect of NaB was fully abolished only when T007 1 μM (a selective PPARγ antagonist) was used in C2C12 cells silenced for GPR109A (Fig 7A and B), thus indicating that NaB exerts its anti‐inflammatory action in muscle cells through the concomitant activation of GPR109A and PPARγ. Moreover, in C2C12 cells not exposed to LPS, we found that NaB, also in this case in a manner depending on GPR109A and PPARγ activation, stimulates autophagy (Fig 7C).

**Figure 8:**
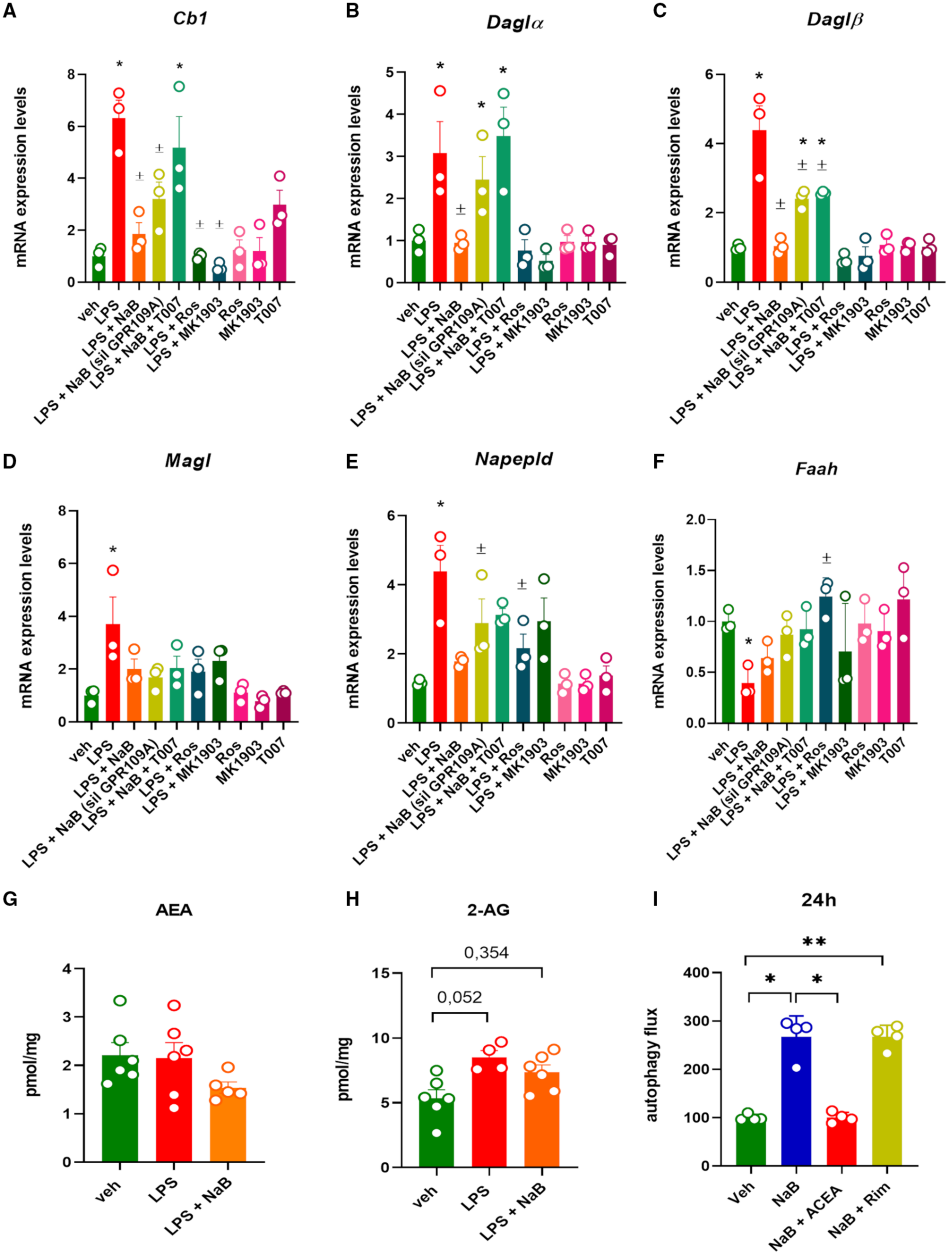
Effect of LPS on the endocannabinoid system activity in C2C12 cells A–FBar chart with individual points showing the mRNA expression levels of *Cb1*, *Daglα*, *Daglβ*, *Magl*, *Napepld*, and *Faah* in control (vehicle, DMSO) and/or GPR109A‐silenced C2C12 myoblasts exposed to LPS (1 μg/ml) in the presence or absence of either MK1903 (1 μM), rosiglitazone (1 μM), or T007 (1 μM).G, HLevels of AEA and 2‐AG were measured in C2C12 cells exposed to LPS (1 μg/ml) or NaB (3 mM) for 24 h.IEffect of ACEA (1 μM) and rimonabant (1 μM) on autophagosome formation measured in C2C12 cells. Data Information: Each bar is the mean ± S.E.M. from 3 independent biological replicates. **P ≤ 0.005; *P ≤ 0.05 vs. the veh group; ^±^ *P* ≤ 0.05 vs. the LPS group calculated using ANOVA.

Next, we evaluated whether NaB could then regulate dysfunctional ECS activity also in myoblasts. In agreement with previous studies, we found that LPS significantly altered the expression of key genes regulating ECS activity (Turcotte *et al*, 2015). Among them, the mRNA expression of *Cb1*, *Daglα*, *and Daglβ* (the latter two are genes encoding for key enzymes producing 2‐AG), *Magl* (2‐AG degradation), and *Napepld* (AEA synthesis) was significantly increased. By contrast, the expression of *Faah* (AEA degradation) was reduced by LPS (Fig 8A–F). Pretreatment with NaB for 30 min before the stimulation with LPS prevented the dysregulated expression of all the aforementioned genes (Fig 8A–F). The effect of NaB was, with the only exception of *Magl*, abolished upon silencing of GPR109A and/or in presence of T007 (Fig 8A–F) and mimicked by rosiglitazone (1 μM) and MK1903 (1 μM) (Fig 8A–F). The drug alone (rosiglitazone, MK1903, and T007 for 3 h) did not significantly change ECS gene expression (Fig 8A–F). Additionally, following stimulation with LPS in C2C12 cells, we did not detect significant changes in AEA levels, although a tendency to reduce its levels was observed following NaB treatment (Fig 8G). By contrast, 2‐AG levels tended to be increased by LPS (Fig 8H). Notably, we also found that the promotion of autophagy in C2C12 cells induced by NaB was abolished in the presence of ACEA, but not rimonabant (Fig 8I).

### Butyrate prevents LPS‐mediated down‐regulation of microRNA (miRNAs) targeting CB1

**Figure 9:**
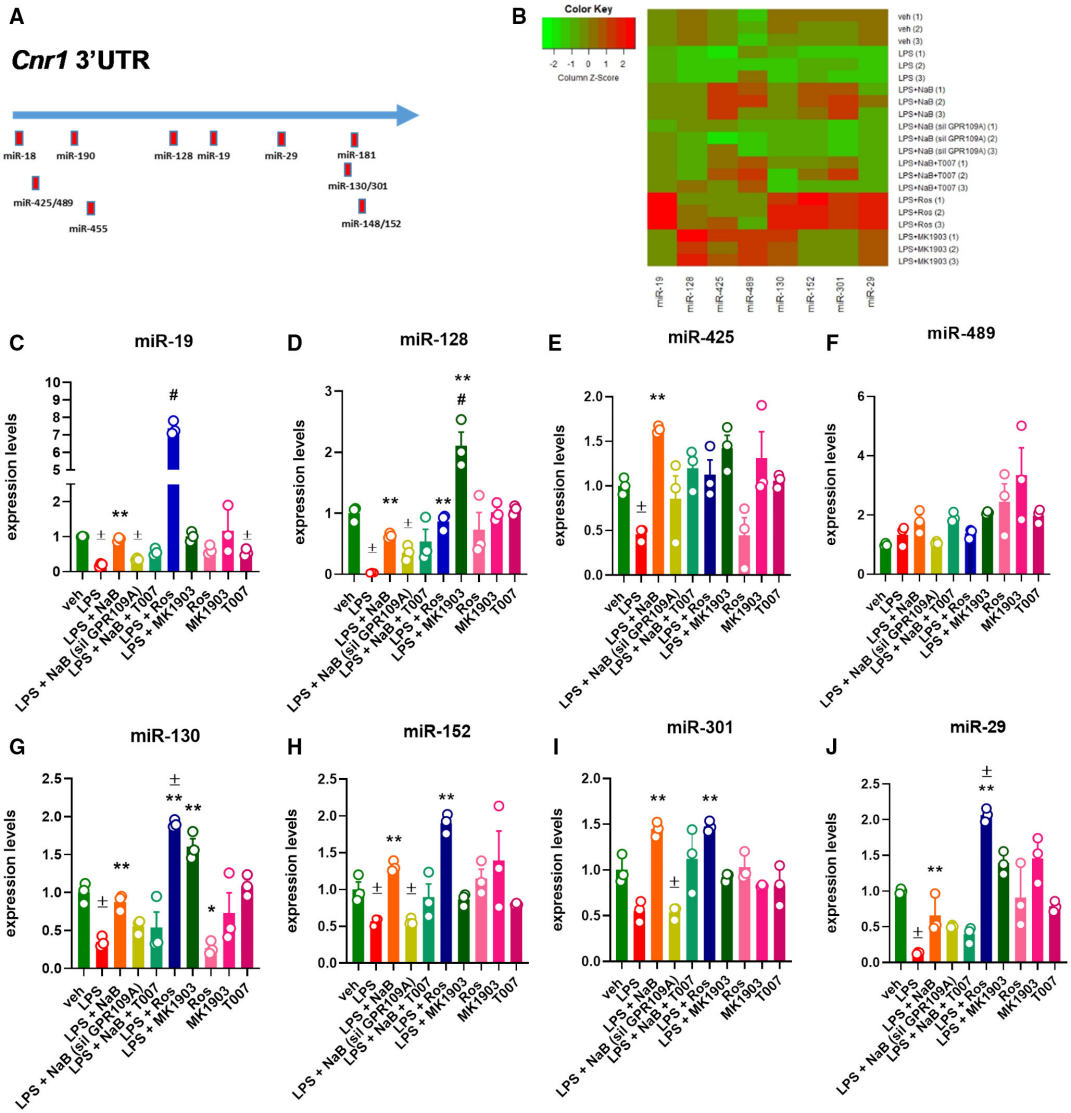
Effect of LPS on the expression of miRNAs targeting the *Cnr1* gene ASchematic representation of miRNAs targeting the 3′‐UTR region of both murine and human CB1 gene.BHeatmap representation of the expression of selected miRNAs in the indicated biological replicates. Red—up‐regulated; green—down‐regulated.C–JBar chart with individual points showing the expression of selected miRNAs in control and Gpr109A‐silenced C2C12 myoblasts exposed to LPS (1 μg/ml) in the presence or absence of either NaB (3 mM), MK1903 (1 μM), or rosiglitazone (1 μM). NaB was also tested in the presence or absence of either rosiglitazone (1 μM) or T007 (1 μM). Data Information: Each bar is the mean ± S.E.M. from 3 independent biological replicates. ^±^ *P* ≤ 0.05 vs. veh group; **P ≤ 0.03 vs. LPS group; ^#^ *P* ≤ 0.05 vs. the other experimental groups calculated using ANOVA.

We next searched for the molecular mechanism through which the stimulation of GPR109A and PPARγ receptors by NaB prevents LPS‐induced dysregulation of CB1, the main effector of the ECS. Therefore, using bioinformatics analysis, we identified several microRNA (miRNAs) sequences targeting the three prime untranslated regions (3′‐UTR) of the *Cb1* (*Cnr1*) murine gene. In particular, we focussed mostly on those conserved among mammals such as miR‐18, miR‐190, miR‐128, miR‐19, miR‐29, miR‐181, miR‐130, miR‐301, miR‐148, and miR‐152 (Fig 9A). However, our analysis also included miR‐429, miR‐489, and miR‐452, which target the murine 3′UTR *Cb1* region, but not the human one. Subsequently, the expression of selected miRNAs was evaluated in C2C12 myoblasts exposed to LPS in the presence or absence of NaB, rosiglitazone, or MK1903. Using quantitative PCR, we found that in myoblasts only miR‐19, miR‐128, miR‐425, miR‐489, miR‐130, miR‐152, miR‐301, and miR‐29 were expressed. As shown in the heatmap (Fig 9B) and bar graphs (Fig 9C–J), the expression of all these miRNAs (except for miR‐489) was significantly reduced by LPS. In cells exposed to LPS in the presence of NaB, we observed that the LPS‐induced down‐regulation of miR‐19, miR‐128, miR‐425, miR‐130, miR‐152, miR‐301 and miR‐29 was prevented, with the expression of miR‐425 being significantly increased compared with the veh group (Fig 9C–J). The effect of NaB was, to a different extent, prevented in C2C12 silenced for *Gpr109A* or by T007 (1 μM). Accordingly, in cells exposed to LPS in the presence of rosiglitazone, we observed that miR‐19, miR‐130, miR‐152, and miR‐29 expression was significantly increased compared with both LPS alone and veh groups (Fig 9C, G, H, and J). Additionally, the expression of miR‐128, miR‐425, and miR‐301 was restored to levels comparable to those of the control (veh) group (Fig 9D, E, and I). Moreover, in cells exposed to LPS in the presence of MK1903, we found that the expression of miR‐128, miR‐425, and miR‐130 was significantly increased versus both the LPS and veh groups (Fig 9D, E, and G), whereas the expression of miR‐19, miR152, miR‐301, and miR‐29 was rescued to control levels (Fig 9C, H, I, and J). Instead, no significant effects were found in cells exposed to the drugs alone, with the only exception of miR‐19 and miR‐130 expression, which was reduced compared to that of the veh group by T007 and rosiglitazone, respectively (Fig 9C–J).

In summary, these results show that NaB in skeletal muscle cells exposed to LPS exerts anti‐inflammatory and pro‐autophagy effects and concomitantly prevents the dysregulated expression of key genes regulating ECS activity through a mechanism depending sequentially on GPR109A and PPARγ activation and, for CB1 receptor expression, miRNA regulation.

### Butyrate protects primary myoblasts isolated from DMD donors from inflammation and impaired autophagy

**Figure 10:**
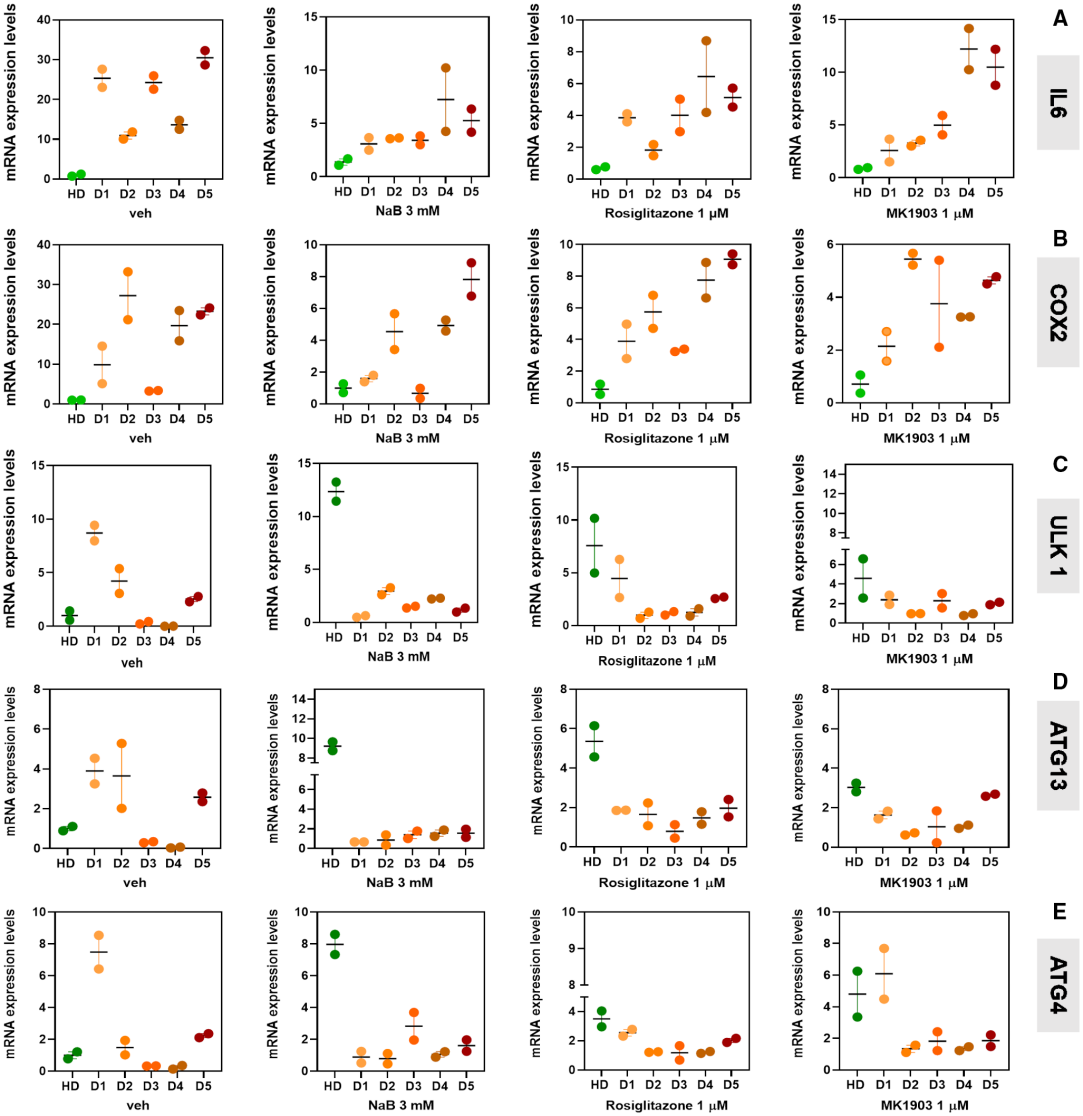
Effect of NaB, MK1903, and rosiglitazone in primary myoblasts isolated from DMD donors A–EBar chart showing the mRNA expression levels of IL6, COX2, ULK 1, ATG13, and ATG4 mRNA in primary human myoblasts isolated from one healthy donor (HD) and five DMD donors (D1–D5). Data Information: The quantification of transcripts by quantitative real‐time PCR was measured twice for each sample.

Finally, we evaluated the effect of NaB, as well as of GPR109A and PPARγ activation by MK1903 and rosiglitazone, respectively, on the expression of inflammatory (*IL6* and *COX2*) and autophagy (*ULK1*, *ATG13*, *ATG4*) genes in primary myoblasts isolated from muscle biopsies of young patients diagnosed with DMD (D1‐D5) caused by different mutations in the dystrophin gene (see Table EV2). Indeed, the expression of both inflammatory and autophagy genes observed in myoblasts from five DMD patients (D1‐D5) was dysregulated when compared to control cells obtained from healthy donors (HD). This dysregulation was largely, albeit not entirely, prevented following treatment with NaB (3 mM), MK1903 (1 μM), and rosiglitazone (1 μM) for 24 h (Fig 10A–E).

## Discussion

In recent decades, interest in the gut microbiota and its related metabolites has rapidly flourished owing to their prominent role in contributing to the overall health of the host. Gut microbial dysbiosis is, therefore, associated with the pathogenesis and/or progression of a broad spectrum of metabolic, neurological, and inflammatory disorders (Gentile *et al*, 2020; Morais *et al*, 2020). In line with this, increasing evidence shows that the therapeutic transplantation of fecal bacteria or the use of pro‐/prebiotic products can lead to future novel opportunities to treat numerous diseases through the correction of gut microbiota imbalance (i.e., dysbiosis) (Kho & Lal, 2018). Nutritional, metabolic and gastrointestinal problems frequently occur in patients with DMD (Pane *et al*, 2006; Brumbaugh *et al*, 2018). In this regard, it has been estimated that chronic persistent inflammation, forced sedentariness, and long‐term use of anti‐inflammatory steroid drugs cause overweight and obesity in more than half of the children with DMD. On the contrary, adolescents and adults with DMD frequently experience underweight due to swallowing dysfunction, lack of dietary fiber, and reduced intestinal motility (Kraus *et al*, 2016; Brumbaugh *et al*, 2018). Similar pathological features were found also in mdx mice, a widely used experimental model of DMD (Mulè *et al*, 2010; Radley‐Crabb *et al*, 2011). Thus, based on this background, in this study, we explored whether the gut microbiota is implicated in the development and progression of DMD. With this aim, we first performed a 16S rRNA Gene Sequencing analysis to characterize the type and relative abundance of bacterial taxa in fecal samples of wild‐type and mdx mice. Our analysis revealed that dystrophic mice are characterized by a higher abundance of the *Prevotellaceae* family, whereas, on the contrary, the relative abundance of the *Saccharimonadaceae*, *Helicobacteriaceae*, *Peptococcaceae*, and *Clostridiales_vadinBB60* families was reduced. A plausible explanation of these changes might be provided by previous data showing that members of the *Prevotellaceae* family (which includes approximately 40 different species) rapidly grow and divide in inflammatory microenvironments like those caused, for instance, by colorectal cancer, arthritis, and inflammatory bowel diseases (Kleessen *et al*, 2002; Lucke *et al*, 2006; Hofer, 2014). On the contrary, Ortega‐Hernández *et al* (2020) recently documented that *Saccharimonadaceae* abundance is negatively correlated with the development of metabolic dysfunctions (Ortega‐Hernández *et al*, 2020). Changes in the abundance of *Peptococcaceae*, *Helicobacteraceae*, *Desulfovibrionaceae*, *Erysipelotrichaceae*, and *Clostridiales* observed here could be instead attributed to reduced physical activity (Liu *et al*, 2015). Importantly, DFZ, a standard of care for the treatment of DMD, improved several pathological features of the disease in mdx mice and concomitantly rescued *Prevotellaceae*, *Saccharimonadaceae*, and *Clostridiales_vadinBB60* levels, suggesting that alterations of these gut bacterial families might be involved in these features. DFZ also altered the relative abundance of families that were not altered in mdx mice, i.e., *Desulfovibrionaceae* and *Erysipelotrichaceae*, which were, respectively, increased and reduced.

Irrespective of the underlying cause(s), we addressed next the question of the potential functional importance of the changes in gut microbiota composition in mdx mice, reported here for the first time. The physiological and pathological function of the gut microbiome is largely mediated by the specific metabolites that this multitude of microorganisms produce. SCFAs, and butyrate in particular, are among the most studied such metabolites, and we found here that their levels in the plasma were, or tended to be, reduced in mdx mice and elevated by DFZ, concomitantly with the therapeutic action of the latter drug. It is known that the main butyrate‐producing bacteria in the gut belong to the phylum *Firmicutes* (Parada Venegas *et al*, 2019). However, other species from the *Bacteroidetes*, *Patescibacteria*, and *Proteobacteria* phyla produce butyrate and the other SCFAs measured here^,^(Alkadhi *et al*, 2014; Cheng *et al*, 2018; Russell *et al*, 2019; Morya *et al*, 2020). Therefore, it is tempting to hypothesize that the reduced SCFA levels observed in the plasma of mdx mice might be due to the lower abundance of *Peptococcaceae* and *Clostridioles_vadin BB60* (*Firmicutes*), *Saccharimonadaceae* (*Patescibacteria*), and *Helicobacteriaceae* (*Proteobacteria*), which were decreased, but not to the increased abundance of *Prevotellaceae* (*Bacteroidetes*). Conversely, the increased SCFA levels observed in DFZ‐treated mdx mice could be related to the increased abundance of *Saccharimonadaceae* and *Clostridiales* but also of a family that was not modified by the presence of dystrophy alone, i.e., *Desulfovibrionaceae* (*Proteobacteria*) (Alkadhi *et al*, 2014; Bosman *et al*, 2019; Morya *et al*, 2020), and not to *Erysipelotrichaceae* (*Firmicutes*) or *Prevotellaceae*, which were both decreased by the drug. Future studies employing shotgun metagenomics approaches and microbiota transfer experiments will be needed to fully understand what species are responsible for the observed changes in SCFAs in mdx mice, with or without treatment with DFZ. Additionally, it will also be interesting to investigate why SCFA levels were found here to be reduced only in the plasma, and not in the skeletal muscle or feces, of mdx mice. As reported in several other studies, SCFAs are produced at varying ratios, with acetate being the most abundant in the colon (~ 60%), followed by propionate (~ 25%), and butyrate (~ 15%) (Duncan *et al*, 2004), which may explain why, in plasma, we could not detect this latter metabolite when using a less sensitive analytical technique (NMR). Moreover, colonic SCFAs are largely utilized by colonocytes as an energy source. The remaining SCFAs reach the liver where they are metabolized, oxidized, or used as a substrate for gluconeogenesis and lipogenesis (Boets *et al*, 2017), which may explain why in plasma we could only detect hydroxyl‐butyrate with NMR. As a consequence, only SCFAs that are not processed by the liver end up in peripheral circulation and eventually in peripheral tissues such as the skeletal muscle (Richards *et al*, 2016). Thus, colonic absorption and hepatic metabolism of SCFAs might have masked differences in the concentrations of these metabolites in the muscle or feces.

As the next step in our study, we investigated the potential relationship between defective circulating SCFAs and autophagy and inflammation in the skeletal muscle of dystrophic mice and/or C2C12 cells. Increased inflammation and decreased autophagy are two hallmarks of DMD muscles, and both play a key role in the progression of this disorder (De Palma *et al*, 2014). Previous reports have shown that SCFAs (and KBs, which we also found here to be decreased in mdx mouse plasma) are intimately connected to both autophagy and inflammation, although there is only a limited number of studies investigating the signaling mechanisms underlying these actions (Tang *et al*, 2011; Rojas‐Morales *et al*, 2016; Feng *et al*, 2018). Here, we focussed our attention on butyrate as the SCFA whose mechanism of action has been perhaps most investigated in previous studies (Walsh *et al*, 2015; Gao *et al*, 2019) and found that this metabolite, administered for 3 weeks to mdx mice up to 19 weeks of age, is capable, like DFZ, of reducing locomotor impairment as well as skeletal muscle inflammation and autophagy deficits. Current evidence indicates that SCFAs exert their effects through three major mechanisms involving: (i) activation of G protein‐coupled receptors (GPCRs); (ii) activation of PPARγ receptors; and (iii) inhibition of histone deacetylases (HDACs) (Dalile *et al*, 2019). We have shown here, to our knowledge for the first time, that NaB, similarly to MK1903 (a selective GPR109A agonist) and rosiglitazone (a selective PPARγ agonist), counteracts LPS‐evoked inflammation and promotes autophagy in murine myoblasts. Noteworthy, this effect was partly abolished in GPR109A‐silenced cells, but fully abolished in these same cells in the presence also of T007 (a selective antagonist of PPARγ), pointing to the participation of both GPR109A and PPARγ in the mechanism of action of NaB, and arguing against inhibition of histone deacetylation as a potentially residual mechanism. Most importantly, the preservation of autophagy and the anti‐inflammatory effect of NaB, MK1093, and rosiglitazone were also observed in primary human myoblasts isolated from DMD donors.

SCFAs are also known to cross the blood–brain barrier (BBB) and modulate neuroendocrine stress reactivity (Dalile *et al*, 2020). However, to date, only a few studies investigated the role of SCFAs in the hypothalamic–pituitary–adrenal (HPA) axis reactivity. One study found that, in rats, high doses of NaB (1.2 g/kg) acted as a pharmacological stressor, increasing plasma levels of the stress markers corticosterone and adrenocorticotropic hormone (ACTH) (Gagliano *et al*, 2014). By contrast, others found that a low dose of NaB (200 mg/kg) only slightly increased ACTH (Dalile *et al*, 2020). In addition, Van de Wouw et al found that oral administration of a cocktail of SCFAs (67.5 mM acetate, 25 mM propionate, and 25 mM butyrate) for 1 week in mice ameliorated stress‐induced corticosterone potentiation after an acute stressor (van de Wouw *et al*, 2018). Therefore, we cannot exclude a no matter how minimal effect of NaB 100 mg/Kg, which is the dose used in this study, on the HPA axis in mdx mice. Further studies are needed to clarify this point.

Endocannabinoid signaling at CB1 is involved in DMD onset and progression (Iannotti *et al*, 2018) and has been suggested to cross‐talk with the gut microbiome (Cani *et al*, 2016; Manca *et al*, 2020). We previously demonstrated that the expression of CB1 receptors is increased in skeletal muscles of both 5‐week (disease onset) and 8‐week‐old mdx mice (Iannotti *et al*, 2018). Here, in skeletal muscles of 19‐week‐old mdx mice, characterized by strong inflammation and reduced autophagy, we found that, along with increased CB1 and CB2 mRNA and protein expression, also the plasma levels of the CB1 endogenous agonist, AEA, were remarkably higher. These findings, therefore, confirmed that the ECS is overactive also in mice with advanced muscular dystrophy. Since we previously demonstrated by RNA seq analysis performed in skeletal muscle tissues that CB1 is differentially expressed in satellite, myoblast, and myotube cells, while on the contrary, CB2 expression is mainly restricted to skeletal muscle‐resident macrophages (Iannotti *et al*, 2018), we focused our attention on CB1 receptors. Our results show that the dysregulated expression of autophagy‐related genes found in 19‐week‐old mdx mice is restored to physiological levels by counteracting ECS overactivity through antagonism of CB1 receptors with rimonabant, whereas CB1 activation by ACEA exacerbated the decreased expression of such genes. It is worth mentioning that previous studies demonstrated that CB1 receptor stimulation leads to the activation of the pAkt/mTOR pathway, a well‐known signaling pathway leading to the inhibition of autophagy (Gómez del Pulgar *et al*, 2000).

Notably, in these mice, the dysregulation of ECS activity was prevented by the administration of DFZ and NaB. This finding suggests that ECS overactivity, which plays a crucial role in the etiopathology of muscular dystrophy (Iannotti *et al*, 2018): (i) exerts this pathological action also by causing defective autophagy in the skeletal muscle, with ensuing exacerbation of the disorder, and (ii) is due, at least in part, to defective butyrate production by the gut microbiota, an effect reversed by DFZ.

Using again C2C12 cells, we next demonstrated that, in a manner dependent on GPR109A and PPARγ, NaB prevents the dysregulated expression of key genes regulating endocannabinoid activity and levels under basal conditions. Notably, the stimulatory effects of NaB on autophagy in these cells were obstructed by the CB1 agonist ACEA. This suggests that, while in healthy mice gut microbiota activity contributes to muscle functionality and regeneration through mechanisms depending on the production and release of SCFAs and consequent activation of GPR109A and PPARγ receptors, in dystrophic mice, the altered gut microbiota composition leads to inadequate production and circulation of butyrate, which causes excessive endocannabinoid system activity at CB1 receptors. This latter condition then participates in exacerbating inflammation and impairing muscle autophagy. It is worth mentioning that, in agreement with our proposed PPARγ‐mediated down‐regulation of ECS overactivity by butyrate, previous studies in preadipocytes and adipocytes have reported that PPARγ activation down‐regulates both endocannabinoid levels and CB1 receptor expression (Matias *et al*, 2006; Pagano *et al*, 2007). Conversely, no previous evidence exists suggesting that GPR109a down‐regulates ECS signaling. Here, by combining computational and experimental approaches, we found that microRNA sequences (miRNAs) targeting the *Cnr1* mRNA 3′UTR region were down‐regulated following C2C12 exposure to LPS. The miRNAs are a class of noncoding RNAs playing a key role in regulating the expression of target genes. In the majority of cases, miRNAs interact with the 3′ UTR region of target mRNAs to induce their degradation and/or translational repression, thus affecting a multitude of biological processes including cell proliferation, differentiation, and survival (O'Brien *et al*, 2018). We found that in C2C12 myoblasts, LPS causes the down‐regulation of miR‐19, miR‐128, miR‐425, miR‐130, miR‐152, miR‐301, and miR‐29. Remarkably, the expression of most of these CB1‐down‐regulating miRNAs was variedly restored, or even further increased, in the presence of NaB, rosiglitazone, or MK1903. This finding suggests that butyrate exerts its protective down‐regulatory function on inflammation‐increased CB1 receptor expression (which is deleterious to skeletal muscle, as recently demonstrated also by others; Haddad, 2021) via the GPR109A‐ and PPARγ‐mediated up‐regulation of *Cnr1*‐targeting miRNAs.

In conclusion, we have reported here a novel mechanism by which gut dysbiosis associated with late‐stage muscular dystrophy in mdx mice may participate in some of the features of this disorder through the reduced release of SCFAs in the blood and impaired GPR109A and PPARγ activation in skeletal muscle, with subsequent disinhibition of endocannabinoid signaling at CB1 receptors and exacerbation of inflammation and autophagy deficit in this tissue. Importantly, we have also shown that butyrate, as well as GPR109A and PPARγ activation, counteract impaired autophagy and inflammation also in myotubes isolated from DMD patients. The finding of this new example of gut microbiome‐endocannabinoid system axis dysregulation [see (Cani *et al*, 2016) for review] may offer the opportunity to treat DMD using gut microbiota‐targeted strategies, on top of the current often poorly effective or unsafe treatments.

## Materials and Methods

### Animal model and drug treatment

The Animal Study Protocol (IACUC; 536/2018) was approved by the Italian Ministry of Health and Ethics Committee for the use of experimental animals being conformed to guidelines for the safe use and care of experimental animals following the Italian D.L. no. 116 of 27 January 1992 and associated guidelines in the European Communities Council (86/609/ECC and 2010/63/UE). In this study, 5‐week‐old control (C57BL/10ScSnJ) and dystrophic (C57BL/10ScSn‐DMDmdx/J) mice weighing approximately 20–25 g were purchased from Charles River Laboratories (Milan IT). All mice were housed in an individually ventilated cage system with a 12‐h light–dark cycle and received standard mouse chow (Harlan Teklad) and water *ab libitum*. Animals belonging to each cage were randomly assigned to the different experimental groups. Each experimental group included at least five mice. The experimenter(s) performing the treatments and locomotor testing was blind to the genotype and treatment. Control or mdx mice were treated orally for 3 weeks with (i) vehicle (dimethyl sulfoxide – DMSO Cat# 276855 Sigma‐Aldrich), (ii) deflazacort (DFZ) 1.2 mg/kg/day (Cat# SML0123 Sigma‐Aldrich), (iii) sodium butyrate (NaB) 100 mg/kg/day (Cat# 303410, Sigma‐Aldrich); (iii) ACEA 2.5 mg/Kg (Cat# A9719 Sigma‐Aldrich), or (iv) rimonabant 0.5 mg/Kg (Cat# 9000484, Cayman) were intraperitoneally (IP) injected three times a week for 2 weeks (Iannotti *et al*, 2018).

### Rotarod test

The rotarod test was performed in control and dystrophic mice at the end of the pharmacological treatment. Briefly, the rotarod was settled with a start speed of 5 rpm, and the mice were placed on the rotating rod for 30 s. Then, the rotarod was accelerated to 40 rpm in 240 s. The time (s) when mice dropped from the rod was recorded. The results were expressed as an average of two different trials, and the interval time of each trial was 30 min (Iannotti *et al*, 2018).

### Muscle strength test

To test the forelimb strength of dystrophic mice treated or not with DFZ and NaB, four weights of 20, 33, 46, and 59 g were used. Mice were handled by the base of the tail and were allowed to grip the first weight (20 g) and to hold 3 s was the criterion. If the mouse dropped the weight in less than 3 s, we tried the same weight again for a maximum of three times. If the mouse held it for 3 s, then we tried it on the next heaviest weight. The mouse was assigned the maximum time/weight achieved. The final total score is calculated as the product of the number of links in the heaviest chain held for the full 3 s, multiplied by the time (s) it is held (Iannotti *et al*, 2018).

### DNA extraction and 16S rRNA gene sequencing

DNA was extracted from fecal samples using the QIAmp PowerFecal DNA kit (Qiagen, Hilden, Germany) according to the manufacturer's instructions. The DNA concentrations of the extracts were measured fluorometrically with the Quant‐iT PicoGreen dsDNA Kit (Thermo Fisher Scientific, MA, USA), and the DNAs were stored at −20°C until 16S rDNA library preparation. Briefly, 1 ng of DNA was used as a template, and the V3‐V4 region of the 16S rRNA gene was amplified by polymerase chain reaction (PCR) using the QIAseq 16S Region Panel protocol in conjunction with the QIAseq 16S/ITS 384‐Index I (Sets A, B, C, D) kit (Qiagen, Hilden, Germany). The 16S metagenomic libraries were eluted in 30 μl of nuclease‐free water, and 1 μl was qualified with a Bioanalyser DNA 1000 Chip (Agilent, CA, USA) to verify the amplicon size (expected size ~ 600 bp) and quantified with a Qubit (Thermo Fisher Scientific, MA, USA). Libraries were then normalized and pooled to 2 nM, denatured, and diluted to a final concentration of 6 pM. Sequencing (2 × 300 bp paired‐end) was performed using the MiSeq Reagent Kit V3 (600 cycles) on an Illumina MiSeq System. Sequencing reads were generated in less than 65 h. Image analysis and base calling were carried out directly on the MiSeq. Data were processed using the DADA2 pipeline (Callahan *et al*, 2016), and taxonomic assignation was performed against the SILVA 132 rRNA reference database (Quast *et al*, 2013). Relative microbiota abundances were obtained by Cumulative Sum Scaling (CSS, MetagenomeSeq R package) (Paulson *et al*, 2013), and microbiota composition was assessed by calculating α‐ and β‐diversity indexes and intra‐ and inter‐individual variations in microbial composition using PERMANOVA (vegan R package).

### Feces samples collection and SCFAs quantification

For fecal collection, mice were put in clean empty cages (without bedding and/or nestle) in the morning around 7:30–8 am (mice have better intestinal transit in the morning). When the last cage is changed, the first mice should have dropped their first feces. Feces were collected with sterile forceps. Samples were stored at −80°C until analysis. For SCFA extraction and measurement by gas chromatography, feces were dissolved in water and suspensions were homogenized for 2 min with a Bead Ruptor 12 (Omni International, Kennesaw, GA, USA) and then centrifuged at 18,000 *g* for 10 min at 4°C. The supernatant was collected and spiked with a solution containing an internal standard (4‐methyl valeric acid) and H3PO4 10% to obtain a pH of about 2. A volume of methyl tert‐butyl ether equivalent to the volume of the diluted sample was added and mixed by vortexing for 2 min. Samples were then centrifuged for 10 min at 18,000 *g* at 4°C, and the organic phases were transferred to glass vials. SCFA analysis was performed on a GC‐FID system (Shimadzu), consisting of a GC 2010 Plus gas chromatograph equipped with an AOC‐20s auto‐sampler, an AOC‐20i auto‐injector, and a flame ionization detector. The system was controlled by GC solution software. One microliter of the organic phase was injected in a split mode into a Nukol capillary GC column (30 m × 0.25 mm id, 0.25 μM film thickness, Supelco analytical), and hydrogen was used as the carrier gas. The injector and detector were set at 250°C. The oven temperature was initially programmed at 60°C, then increased to 200°C at 12°C/min, and held for 2 min. SCFAs were quantified using a 5‐point calibration curve prepared with a mix of standards (acetic acid, propionic acid, butyric acid, isobutyric acid, valeric acid, and isovaleric acid) extracted following the same procedure as samples.

### GC–MS analysis

Plasma was analyzed by gas chromatography–mass spectrometry (GC ‐ 7890A, Agilent Technologies; MS ‐ 5977A MSD, Agilent Technologies). In brief, 100 μl of plasma was diluted with 900 μl of saline. 500 μl of this solution was added to 20 μl of H_3_PO_4_ 85% (w/v) and vortexed for 5 min. Then, to each sample, 500 μl of diethyl ether was added. The suspension was vortexed for 5 min and centrifuged at 14,000 rpm for 30 min at room temperature. After the supernatant was taken and sodium sulfate anhydrous was added. Finally, the organic phase was placed in a new glass tube for GC–MS analysis. The GC was programmed to achieve the following run parameters: initial temperature of 90°C, hold for 2 min, a ramp of 2°C/min up to a temperature of 100°C, hold of 10 min, and ramp of 5°C/min up to a final temperature of 110°C for a total run time of 21 min.

### Metabolites extraction from tissues

To extract metabolites, tissues were mechanically disrupted. We used the methanol/water/chloroform protocol as suggested (Lindon *et al*, 2005). After solvent removal with a rotary vacuum evaporator at room temperature, samples were stored at −80°C until analysis.

### NMR measurements of polar metabolites

Polar fractions of muscle samples were re‐suspended in 630 μl of phosphate buffer saline (PBS, pH 7.4 Cat# D8537) and 70 μl of ^2^H_2_O solution [containing 1 mM sodium 3‐trimethylsilyl [2,2,3,3‐^2^H_4_] propionate (TSP) was added as a chemical shift reference and assumed to resonate at δ = 0.00 ppm] to provide a field frequency lock. One‐dimensional (1D) spectra were acquired at 27°C on a Bruker Avance III‐600 spectrometer operating at 600.13 MHz and equipped with a TCI CryoProbe™, using the excitation‐sculpting sequence for solvent suppression (Hwang & Shaka, 1995). Subsequently, 330 μl of each serum sample was diluted with 300 μl of saline solution with 0.9% sodium chloride (pH 7.4) and 70 μl of ^2^H_2_O. To strongly attenuate protein signals, T_2_‐edited 1D spectra were collected using short spin–spin relaxation times in the Carr‐Purcell‐Meiboom‐Gill (CPMG) pulse sequence with water presaturation (de Graaf & Behar, 2003) and using a fixed inter‐echo delay to eliminate diffusion and J‐modulation effects. Two‐dimensional (2D) clean total‐correlation spectroscopy (TOCSY) and heteronuclear single‐quantum coherence (HSQC) experiments were also acquired for metabolite identification. 2D spectra were referenced to the lactate doublet assumed to resonate at δ = 1.33 ppm for ^1^H and δ = 20.76 ppm for ^13^C. Metabolites were identified by comparison with an online database (Wishart *et al*, 2018).

### NMR data processing and multivariate statistical analysis

The 0.50–9.50 ppm region of the proton spectra of mice muscle (gastrocnemius) was automatically segmented into integrated regions (buckets) of 0.02 ppm each using the AMIX 3.6 package (Bruker Biospin, Germany). The 4.55–5.15 ppm region around the water resonance was excluded, and the binned regions were normalized to the total spectrum area. For sera, we used the selected 0.60–8.60 ppm spectral area, and spectra were analyzed as above, excluding the 4.46–5.16 ppm water resonance region. Multivariate statistical data analysis was applied to the muscle and serum dataset to differentiate treated/untreated mdx and healthy profiles according to their metabolic content. Each dataset was reshaped as a matrix and imported into SIMCA 14 package (Umetrics, Umea, Sweden), where unsupervised PCA followed by supervised OPLS‐DA discriminant analyses was performed (Eriksson *et al*, 2006). PCA was first applied to check outliers and uncover trends and clusters, while OPLS‐DA was used to improve group discrimination. Moreover, O2PLS analysis was performed to generate a bilinear and joint model for NMR and gut microbiota data. We also generated correlation maps with hierarchical clustering by combining microbiota families values and selected polar metabolite buckets considering Euclidean distance for the metrics and the WARD method for clustering criterion. The performance of each multivariate model was evaluated via R^2^ (the goodness of fit) and Q^2^ (the goodness of prediction) parameters. Each model was validated by a 7‐round internal iterative cross‐validation routine, permutation test response (800 repeats), and analysis of variance (ANOVA testing of cross‐validated predictive residuals). Selected and isolated signals with ¦*P*_corr_¦ ≥ 0.7, VIP (variable importance in the projection) > 1 were then considered for univariate statistical analysis and ANOVA test with Bonferroni correction.

### Cell culture and reagents

Murine C2C12 myoblasts were propagated in a growth medium (GM) composed of Dulbecco's modified Eagle's medium (Cat# 11995065; Life Technologies) supplemented with 10% fetal bovine serum (FBS, Cat# 16000044; Life Technologies), 5,000 U/ml penicillin plus 5,000 μg/ml streptomycin (Cat# 15070063; Life Technologies), and 1% l‐glutamine (Cat# A2916801; Life Technologies). Proliferating C2C12 cells were differentiated into myotubes following the exposure to differentiation medium (DM) composed of Dulbecco's modified Eagle's medium supplemented with 2% horse serum heat‐inactivated (Cat# 26050070, Sigma‐Aldrich) for 3 days (McMahon *et al*, 1994). Primary myoblasts were established from muscle biopsies of DMD donors after they had signed informed consent forms and following the guidelines of the G. Gaslini Institute Ethical Committee and according to published procedures (Morosetti *et al*, 2010). The myoblasts were propagated in Full Aneural Medium composed by Dulbecco's modified Eagle's medium (DMEM; Cat# 11995065) supplemented with 15% FBS, 20% Medium 199 (Cat# 12350039), 1% insulin (Cat# A11382II), 1% l‐glutamine (Cat# A2916801), and 15,000 U/ml penicillin plus 5,000 μg/ml streptomycin (Cat# 15070063), FGF (Cat# PHG6015), EGF (Cat# PHG0311). Primary myoblasts were differentiated in myotubes using a commercially available skeletal muscle differentiation medium (Cat# C‐23061, PromoCell, USA) provided by VWR International PBI S.r.l. in the presence or not of NaB 3 mM (Cat# 303410 Life Technologies), MK1903 (Cat# 4622, Tocris UK), or rosiglitazone (Cat# 5325, Tocris UK).

### RNA extraction and quantitative PCR (qPCR)

Total RNA isolation, purification, and cDNA synthesis were performed as described (Iannotti *et al*, 2018). Total miRNA isolation was performed using RNeasy Mini Kit (cat# 217004, Qiagen). Reverse transcription of miRNA was performed using miScript II RT Kit (cat# 218161, Qiagen). Quantitative PCR (qPCR) was carried out in a real‐time PCR system CFX384 (Bio‐Rad) using the SYBR Green PCR Kit (Cat# 1725274, Bio‐Rad for mRNAs; Cat# 218073, Quiagen for miRNAs) detection technique and specific primer sequences reported in Table EV3. Primer sequences for miRNA were provided by Qiagen. Quantitative PCR was performed on independent biological samples ≥4–5 for each experimental group. Also, each sample was amplified simultaneously in quadruplicate in a one‐assay run with a nontemplate control blank for each primer pair to control for contamination or primer‐dimer formation, and the cycle threshold (Ct) value for each experimental group was determined. The housekeeping genes ribosomal protein S16, glyceraldehyde 3‐phosphate dehydrogenase (GAPDH), and U6 (RNU6‐1) were used to normalize the Ct values, using the 2^^−ΔCt^ formula. Differences in mRNAs and miRNAs content between groups were expressed as 2^^−ΔΔCt^, as previously described (Iannotti *et al*, 2018).

### miRNA target prediction

Bioinformatic analysis to predict putative miRNA target sites within the 3′UTR region of both human and murine CB1 gene was performed using the free software TargetScan (http://www.targetscan.org/vert_80/).

### Western blot

Control and mdx mice were previously anesthetized with 75% CO_2_/25% O_2_ and then sacrificed by cervical dislocation. Gastrocnemius was rapidly dissected on ice and kept on dry ice until the whole procedure was completed. Muscle tissues were homogenized in 1x TNE buffer plus 1% (v/v) Triton X‐100 (Cat# T8787, Sigma‐Aldrich) protease Inhibitor (Cat# P8340, Sigma‐Aldrich) and phosphatase Inhibitor Cocktail 2 (Cat# P5726, Sigma‐Aldrich). Lysates were kept in an orbital shaker incubator at 220 rpm at 4°C for 30 min and then centrifuged for 15 min at 13,000 *g* at 4°C. The supernatants were transferred to tubes and quantified by DC Protein Assay (Cat# 5000116, Bio‐Rad, Milan, Italy). Subsequently, protein samples (60–80 μg of total protein) were heated at 70°C for 10 min in 1X LDS Sample Buffer (Cat# B0007, Life Technology) plus 1X sample reducing agent (Cat# B0009, Life Technology) and loaded on 4–12% Bis–Tris Protein Gels (Cat# NW04120, Life Technology) and then transferred the membrane using Trans‐Blot Turbo Mini 0.2 μm PVDF Transfer Packs (Cat# 1704156 Bio‐Rad). The primary antibodies used were (i) rabbit anti‐Akt Antibody (item n. 9272, Cell Signaling Technology USA); (ii) rabbit anti‐phospho Akt (Ser473) (D9E) XP^®^ (Cat# 4060, Cell Signaling Technology USA); (iii) rabbit anti‐LC3 antibody (Cat# 2775, Cell Signaling Technology USA); (iv) rabbit anti‐CB1 (Cat# Y409605, ABM Canada); (v) mouse anti‐CB2 (Cat# WH0001269MI, Merck); and (vi) an anti‐rabbit Cox2 (D5H5) XP^®^ (Cat# 12282, Cell Signaling ‐ USA). An anti‐GAPDH antibody (1D4) (Cat#. NB300‐221; Novus Biologicals) was used to check for equal protein loading. Reactive bands were detected by Clarity Western ECL Substrate (Cat# 1705061 Bio‐Rad). The intensity of bands was analyzed on a ChemiDoc station with Quantity‐one software (Bio‐Rad, Segrate, Italy).

### Cell transfection and LPS treatment

C2C12 myoblasts were plated in 6‐well culture dishes at a confluency of 60%. The next day GPR109A gene silencing was obtained by transfection of predesigned siRNA sequences (Cat# AM16708, Life Technologies) using Lipofectamine^®^ 2000 reagent (Cat# 11668‐027, Life Technologies) according to the manufacturer's instructions. Control cells were transfected with a scrambled siRNA sequence (Cat# AM4642, Life Technologies) as a negative control. At 24 h following transfection, C2C12 were treated with 1 μg/ml lipopolysaccharides (LPS; O111:B4, Sigma‐Aldrich) for 3 h according to published procedures (Frost *et al*, 2003). NaB 3 mM (den Besten *et al*, 2015) (Cat# 303410 Life Technologies), T0070907 1 μM (Cat# 2301, Tocris UK), MK1903 (Cat# 4622, Tocris UK), and rosiglitazone (Cat# 5325, Tocris UK) were preincubated 1 h before the LPS stimulation.

### Autophagy assay

Control and GPR109A silenced C2C12 myoblasts were cultured in 96‐well flat‐bottom black plates with optimal density (2 × 10^4^ cells/well). The day following plating, C2C12 cells were treated with NaB, MK1903, rosiglitazone, and T007 for 24 and 48 h. Autophagosome activity was detected with a specific dye using an autophagy assay kit (Cat# MAK138, Sigma‐Aldrich). The protocol was performed following the manufacturer's instructions. Fluorescence intensity was measured using the Promega GloMax^®^ Plate Reader.

### Measurement of endocannabinoids

Lipids were extracted from plasma (5 ml) and AEA and 2‐AG prepurified and quantified by isotope dilution liquid chromatography–atmospheric pressure chemical ionization–mass spectrometry (LC‐APCI‐MS) as described previously (Annuzzi *et al*, 2010).

### Statistical analysis

All datasets were subjected to outlier identification and subsequent removal, using the ROUT method using GraphPad Prism version 9. D'Agostino‐Pearson or Shapiro–Wilk tests were used to consider the data normal distribution. Normal data were assessed via one‐way analysis of variance (ANOVA) followed by the Tukey's analysis to determine statistically significant differences between two or more independent biological groups. Data are expressed as mean ± SEM of values. Significance was determined as *P* < 0.05.

**The paper explained**

**Problem**

Duchenne muscular dystrophy (DMD) is the most frequent form of genetic disorder characterized by an irreversible degeneration of skeletal muscles. Therefore, the identification of novel translational approaches aimed to halt or delay disease progression remains an important unmet need.

**Results**

In this study, we found that in mdx mice, a validated preclinical model of DMD, the disease is associated with a significant alteration in the gut microbiota composition compared with healthy controls. Along with this alteration, the plasma of mdx mice showed a reduction in the levels of gut microbiota‐related metabolites, the short‐chain fatty acids (SCFAs), and an elevation of those of endocannabinoids. Supplementation with the SCFA, sodium butyrate (NaB), rescued muscle strength and autophagy, and prevented inflammation associated with excessive endocannabinoid signaling at CB1 receptors to the same extent as deflazacort (DFZ), the standard palliative care for DMD. In C2C12 myoblasts stimulated with lipopolysaccharide, a pro‐inflammatory molecule derived from a malfunctioning gut microbiota, NaB exerted anti‐inflammatory effects, promoted autophagy, and prevented dysregulation of microRNAs that keep under negative control the CB1 receptor gene and did so in a manner depending on the activation of GPR109A and PPARγ receptors.

**Impact**

We highlight the translational value of the gut microbiota‐endocannabinoid system cross‐talk as a novel disease‐modifying approach in DMD, with potential benefits also in other muscular dystrophies.

## Author contributions

**Hilal Kalkan:** Formal analysis; investigation. **Ester Pagano:** Formal analysis; investigation. **Debora Paris:** Formal analysis; investigation. **Elisabetta Panza:** Formal analysis; investigation. **Mariarosaria Cuozzo:** Investigation. **Claudia Moriello:** Formal analysis; investigation. **Fabiana Piscitelli:** Formal analysis; investigation. **Armita Abolghasemi:** Investigation. **Elisabetta Gazzerro:** Methodology. **Cristoforo Silvestri:** Formal analysis. **Raffaele Capasso:** Formal analysis. **Andrea Motta:** Formal analysis. **Roberto Russo:** Formal analysis. **Vincenzo Di Marzo:** Conceptualization; funding acquisition; writing – original draft; writing – review and editing. **Fabio Arturo Iannotti:** Conceptualization; funding acquisition; writing – original draft; writing – review and editing.

## Disclosure statement and competing interests

The authors declare that they have no conflict of interest.

## Supporting information



Expanded View Figures PDF

Table EV1

Table EV2

Table EV3

PDF+

## Data Availability

Raw sequencing data of 16S rRNA sequencing are deposited at the following link: https://www.ncbi.nlm.nih.gov/bioproject/913018

## References

[emmm202216225-bib-0001] Alkadhi S , Kunde D , Cheluvappa R , Randall‐Demllo S , Eri R (2014) The murine appendiceal microbiome is altered in spontaneous colitis and its pathological progression. Gut Pathog 6: 25 25002910 10.1186/1757-4749-6-25 PMC4085080

[emmm202216225-bib-0002] Annuzzi G , Piscitelli F , Di Marino L , Patti L , Giacco R , Costabile G , Bozzetto L , Riccardi G , Verde R , Petrosino S *et al* (2010) Differential alterations of the concentrations of endocannabinoids and related lipids in the subcutaneous adipose tissue of obese diabetic patients. Lipids Health Dis 9: 43 20426869 10.1186/1476-511X-9-43 PMC2868848

[emmm202216225-bib-0003] Bäckhed F , Manchester JK , Semenkovich CF , Gordon JI (2007) Mechanisms underlying the resistance to diet‐induced obesity in germ‐free mice. Proc Natl Acad Sci USA 104: 979–984 17210919 10.1073/pnas.0605374104 PMC1764762

[emmm202216225-bib-0004] den Besten G , Bleeker A , Gerding A , van Eunen K , Havinga R , van Dijk TH , Oosterveer MH , Jonker JW , Groen AK , Reijngoud D‐J *et al* (2015) Short‐chain fatty acids protect against high‐fat diet‐induced obesity via a PPARγ‐dependent switch from lipogenesis to fat oxidation. Diabetes 64: 2398–2408 25695945 10.2337/db14-1213

[emmm202216225-bib-0005] Biggar WD , Skalsky A , McDonald CM (2022) Comparing deflazacort and prednisone in Duchenne muscular dystrophy. J Neuromuscul Dis 9: 463–476 35723111 10.3233/JND-210776 PMC9398085

[emmm202216225-bib-0006] Boets E , Gomand SV , Deroover L , Preston T , Vermeulen K , De Preter V , Hamer HM , Van den Mooter G , De Vuyst L , Courtin CM *et al* (2017) Systemic availability and metabolism of colonic‐derived short‐chain fatty acids in healthy subjects: a stable isotope study. J Physiol 595: 541–555 27510655 10.1113/JP272613 PMC5233652

[emmm202216225-bib-0007] Bosman ES , Albert AY , Lui H , Dutz JP , Vallance BA (2019) Skin exposure to narrow band ultraviolet (UVB) light modulates the human intestinal microbiome. Front Microbiol 10: 2410 31708890 10.3389/fmicb.2019.02410 PMC6821880

[emmm202216225-bib-0008] Boursereau R , Abou‐Samra M , Lecompte S , Noel L , Brichard SM (2018) Downregulation of the NLRP3 inflammasome by adiponectin rescues Duchenne muscular dystrophy. BMC Biol 16: 33 29558930 10.1186/s12915-018-0501-z PMC5861675

[emmm202216225-bib-0009] Brown AJ , Goldsworthy SM , Barnes AA , Eilert MM , Tcheang L , Daniels D , Muir AI , Wigglesworth MJ , Kinghorn I , Fraser NJ *et al* (2003) The orphan G protein‐coupled receptors GPR41 and GPR43 are activated by propionate and other short chain carboxylic acids. J Biol Chem 278: 11312–11319 12496283 10.1074/jbc.M211609200

[emmm202216225-bib-0010] Brumbaugh D , Watne L , Gottrand F , Gulyas A , Kaul A , Larson J , Tomezsko J (2018) Nutritional and gastrointestinal management of the patient with duchenne muscular dystrophy. Pediatrics 142: S53–S61 30275249 10.1542/peds.2018-0333G

[emmm202216225-bib-0011] Bushby K , Finkel R , Birnkrant DJ , Case LE , Clemens PR , Cripe L , Kaul A , Kinnett K , McDonald C , Pandya S *et al* (2010) Diagnosis and management of Duchenne muscular dystrophy, part 1: diagnosis, and pharmacological and psychosocial management. Lancet Neurol 9: 77–93 19945913 10.1016/S1474-4422(09)70271-6

[emmm202216225-bib-0012] Callahan BJ , McMurdie PJ , Rosen MJ , Han AW , Johnson AJA , Holmes SP (2016) DADA2: high‐resolution sample inference from Illumina amplicon data. Nat Methods 13: 581–583 27214047 10.1038/nmeth.3869 PMC4927377

[emmm202216225-bib-0013] Cani PD , Plovier H , Van Hul M , Geurts L , Delzenne NM , Druart C , Everard A (2016) Endocannabinoids — at the crossroads between the gut microbiota and host metabolism. Nat Rev Endocrinol 12: 133–143 26678807 10.1038/nrendo.2015.211

[emmm202216225-bib-0014] Cheng D , Ngo HH , Guo W , Liu Y , Chang SW , Nguyen DD , Nghiem LD , Zhou J , Ni B (2018) Anaerobic membrane bioreactors for antibiotic wastewater treatment: performance and membrane fouling issues. Bioresour Technol 267: 714–724 30082132 10.1016/j.biortech.2018.07.133

[emmm202216225-bib-0015] Cirak S , Feng L , Anthony K , Arechavala‐Gomeza V , Torelli S , Sewry C , Morgan JE , Muntoni F (2012) Restoration of the dystrophin‐associated glycoprotein complex after exon skipping therapy in Duchenne muscular dystrophy. Mol Ther 20: 462–467 22086232 10.1038/mt.2011.248 PMC3277241

[emmm202216225-bib-0016] Crisafulli S , Sultana J , Fontana A , Salvo F , Messina S , Trifirò G (2020) Global epidemiology of Duchenne muscular dystrophy: an updated systematic review and meta‐analysis. Orphanet J Rare Dis 15: 141 32503598 10.1186/s13023-020-01430-8 PMC7275323

[emmm202216225-bib-0017] Cruz‐Guzmán Odel R , Rodríguez‐Cruz M , Escobar Cedillo RE (2015) Systemic inflammation in Duchenne muscular dystrophy: association with muscle function and nutritional status. Biomed Res Int 2015: e891972 10.1155/2015/891972 PMC4561314 26380303

[emmm202216225-bib-0018] Dalile B , Van Oudenhove L , Vervliet B , Verbeke K (2019) The role of short‐chain fatty acids in microbiota–gut–brain communication. Nat Rev Gastroenterol Hepatol 16: 461–478 31123355 10.1038/s41575-019-0157-3

[emmm202216225-bib-0019] Dalile B , Vervliet B , Bergonzelli G , Verbeke K , Van Oudenhove L (2020) Colon‐delivered short‐chain fatty acids attenuate the cortisol response to psychosocial stress in healthy men: a randomized, placebo‐controlled trial. Neuropsychopharmacology 45: 2257–2266 32521538 10.1038/s41386-020-0732-x PMC7784980

[emmm202216225-bib-0020] De Palma C , Perrotta C , Pellegrino P , Clementi E , Cervia D (2014) Skeletal muscle homeostasis in Duchenne muscular dystrophy: modulating autophagy as a promising therapeutic strategy. Front Aging Neurosci 6: 188 25104934 10.3389/fnagi.2014.00188 PMC4109521

[emmm202216225-bib-0021] Di Marzo V (2018) New approaches and challenges to targeting the endocannabinoid system. Nat Rev Drug Discov 17: 623–639 30116049 10.1038/nrd.2018.115

[emmm202216225-bib-0022] Duncan SH , Holtrop G , Lobley GE , Calder AG , Stewart CS , Flint HJ (2004) Contribution of acetate to butyrate formation by human faecal bacteria. Br J Nutr 91: 915–923 15182395 10.1079/BJN20041150

[emmm202216225-bib-0023] Eriksson L , Johansson E , Kettaneh‐Wold N , Trygg J , Wikström C , Wold S (2006) Multi‐ and megavariate data analysis: Part I: Basic principles and applications. Umeå: Umetrics Academy

[emmm202216225-bib-0024] Feng Y , Wang Y , Wang P , Huang Y , Wang F (2018) Short‐chain fatty acids manifest Stimulative and protective effects on intestinal barrier function through the inhibition of NLRP3 inflammasome and autophagy. Cell Physiol Biochem 49: 190–205 30138914 10.1159/000492853

[emmm202216225-bib-0025] Frampton J , Murphy KG , Frost G , Chambers ES (2020) Short‐chain fatty acids as potential regulators of skeletal muscle metabolism and function. Nat Metab 2: 840–848 32694821 10.1038/s42255-020-0188-7

[emmm202216225-bib-0026] Frost RA , Nystrom GJ , Lang CH (2003) Lipopolysaccharide and proinflammatory cytokines stimulate interleukin‐6 expression in C2C12 myoblasts: role of the Jun NH2‐terminal kinase. Am J Physiol Regul Integr Comp Physiol 285: R1153–R1164 12842862 10.1152/ajpregu.00164.2003

[emmm202216225-bib-0027] Gagliano H , Delgado‐Morales R , Sanz‐Garcia A , Armario A (2014) High doses of the histone deacetylase inhibitor sodium butyrate trigger a stress‐like response. Neuropharmacology 79: 75–82 24212060 10.1016/j.neuropharm.2013.10.031

[emmm202216225-bib-0028] Gao F , Lv Y‐W , Long J , Chen J‐M , He J , Ruan X‐Z , Zhu H (2019) Butyrate improves the metabolic disorder and gut microbiome dysbiosis in mice induced by a high‐fat diet. Front Pharmacol 10: 1040 31607907 10.3389/fphar.2019.01040 PMC6761375

[emmm202216225-bib-0029] Gentile F , Doneddu PE , Riva N , Nobile‐Orazio E , Quattrini A (2020) Diet, microbiota and brain health: unraveling the network intersecting metabolism and neurodegeneration. Int J Mol Sci 21: 7471 33050475 10.3390/ijms21207471 PMC7590163

[emmm202216225-bib-0030] Gómez del Pulgar T , Velasco G , Guzmán M (2000) The CB1 cannabinoid receptor is coupled to the activation of protein kinase B/Akt. Biochem J 347: 369–373 10749665 10.1042/0264-6021:3470369 PMC1220968

[emmm202216225-bib-0031] de Graaf RA , Behar KL (2003) Quantitative 1H NMR spectroscopy of blood plasma metabolites. Anal Chem 75: 2100–2104 12720347 10.1021/ac020782+

[emmm202216225-bib-0032] Griggs RC , Miller JP , Greenberg CR , Fehlings DL , Pestronk A , Mendell JR , Moxley RT , King W , Kissel JT , Cwik V *et al* (2016) Efficacy and safety of deflazacort vs prednisone and placebo for Duchenne muscular dystrophy. Neurology 87: 2123–2131 27566742 10.1212/WNL.0000000000003217 PMC5109941

[emmm202216225-bib-0033] Grosicki GJ , Fielding RA , Lustgarten MS (2018) Gut microbiota contribute to age‐related changes in skeletal muscle size, composition, and function: biological basis for a gut‐muscle Axis. Calcif Tissue Int 102: 433–442 29058056 10.1007/s00223-017-0345-5 PMC5858871

[emmm202216225-bib-0034] Haddad M (2021) The impact of CB1 receptor on inflammation in skeletal muscle cells. J Inflamm Res 14: 3959–3967 34421307 10.2147/JIR.S322247 PMC8373309

[emmm202216225-bib-0035] Hillard CJ , Manna S , Greenberg MJ , DiCamelli R , Ross RA , Stevenson LA , Murphy V , Pertwee RG , Campbell WB (1999) Synthesis and characterization of potent and selective agonists of the neuronal cannabinoid receptor (CB1). J Pharmacol Exp Ther 289: 1427–1433 10336536

[emmm202216225-bib-0036] Hofer U (2014) Pro‐inflammatory prevotella? Nat Rev Microbiol 12: 5 24270843 10.1038/nrmicro3180

[emmm202216225-bib-0037] Hwang TL , Shaka AJ (1995) Water suppression that works. Excitation sculpting using arbitrary wave‐forms and pulsed‐field gradients. J Magn Reson A 112: 275–279

[emmm202216225-bib-0038] Iannotti FA , Silvestri C , Mazzarella E , Martella A , Calvigioni D , Piscitelli F , Ambrosino P , Petrosino S , Czifra G , Bíró T *et al* (2014) The endocannabinoid 2‐AG controls skeletal muscle cell differentiation via CB1 receptor‐dependent inhibition of Kv7 channels. Proc Natl Acad Sci USA 111: E2472–E2481 24927567 10.1073/pnas.1406728111 PMC4066524

[emmm202216225-bib-0039] Iannotti FA , Di Marzo V , Petrosino S (2016) Endocannabinoids and endocannabinoid‐related mediators: targets, metabolism and role in neurological disorders. Prog Lipid Res 62: 107–128 26965148 10.1016/j.plipres.2016.02.002

[emmm202216225-bib-0040] Iannotti FA , Pagano E , Guardiola O , Adinolfi S , Saccone V , Consalvi S , Piscitelli F , Gazzerro E , Busetto G , Carrella D *et al* (2018) Genetic and pharmacological regulation of the endocannabinoid CB1 receptor in Duchenne muscular dystrophy. Nat Commun 9: 3950 30262909 10.1038/s41467-018-06267-1 PMC6160489

[emmm202216225-bib-0041] Kho ZY , Lal SK (2018) The human gut microbiome – a potential controller of wellness and disease. Front Microbiol 9: 1835 30154767 10.3389/fmicb.2018.01835 PMC6102370

[emmm202216225-bib-0042] Kleessen B , Kroesen AJ , Buhr HJ , Blaut M (2002) Mucosal and invading bacteria in patients with inflammatory bowel disease compared with controls. Scand J Gastroenterol 37: 1034–1041 12374228 10.1080/003655202320378220

[emmm202216225-bib-0043] Koh A , De Vadder F , Kovatcheva‐Datchary P , Bäckhed F (2016) From dietary fiber to host physiology: short‐chain fatty acids as key bacterial metabolites. Cell 165: 1332–1345 27259147 10.1016/j.cell.2016.05.041

[emmm202216225-bib-0044] Kraus D , Wong BL , Horn PS , Kaul A (2016) Constipation in Duchenne muscular dystrophy: prevalence, diagnosis, and treatment. J Pediatr 171: 183–188 26831528 10.1016/j.jpeds.2015.12.046

[emmm202216225-bib-0045] Kumar J , Rani K , Datt C (2020) Molecular link between dietary fibre, gut microbiota and health. Mol Biol Rep 47: 6229–6237 32623619 10.1007/s11033-020-05611-3

[emmm202216225-bib-0046] Lahiri S , Kim H , Garcia‐Perez I , Reza MM , Martin KA , Kundu P , Cox LM , Selkrig J , Posma JM , Zhang H *et al* (2019) The gut microbiota influences skeletal muscle mass and function in mice. Sci Transl Med 11: eaan5662 31341063 10.1126/scitranslmed.aan5662 PMC7501733

[emmm202216225-bib-0047] Layden BT , Angueira AR , Brodsky M , Durai V , Lowe WL (2013) Short chain fatty acids and their receptors: new metabolic targets. Transl Res 161: 131–140 23146568 10.1016/j.trsl.2012.10.007

[emmm202216225-bib-0048] Le Poul E , Loison C , Struyf S , Springael J‐Y , Lannoy V , Decobecq M‐E , Brezillon S , Dupriez V , Vassart G , Van Damme J *et al* (2003) Functional characterization of human receptors for short chain fatty acids and their role in polymorphonuclear cell activation. J Biol Chem 278: 25481–25489 12711604 10.1074/jbc.M301403200

[emmm202216225-bib-0049] Lindon JC , Nicholson JK , Holmes E , Keun HC , Craig A , Pearce JTM , Bruce SJ , Hardy N , Sansone S‐A , Antti H *et al* (2005) Summary recommendations for standardization and reporting of metabolic analyses. Nat Biotechnol 23: 833–838 16003371 10.1038/nbt0705-833

[emmm202216225-bib-0050] Liu T‐W , Park Y‐M , Holscher HD , Padilla J , Scroggins RJ , Welly R , Britton SL , Koch LG , Vieira‐Potter VJ , Swanson KS (2015) Physical activity differentially affects the Cecal microbiota of ovariectomized female rats selectively bred for high and low aerobic capacity. PLoS One 10: e0136150 26301712 10.1371/journal.pone.0136150 PMC4547806

[emmm202216225-bib-0051] Lucke K , Miehlke S , Jacobs E , Schuppler M (2006) Prevalence of *Bacteroides* and *Prevotella* spp. in ulcerative colitis. J Med Microbiol 55: 617–624 16585651 10.1099/jmm.0.46198-0

[emmm202216225-bib-0052] Manca C , Shen M , Boubertakh B , Martin C , Flamand N , Silvestri C , Di Marzo V (2020) Alterations of brain endocannabinoidome signaling in germ‐free mice. Biochim Biophys Acta Mol Cell Biol Lipids 1865: 158786 32795503 10.1016/j.bbalip.2020.158786

[emmm202216225-bib-0053] Matias I , Gonthier M‐P , Orlando P , Martiadis V , De Petrocellis L , Cervino C , Petrosino S , Hoareau L , Festy F , Pasquali R *et al* (2006) Regulation, function, and dysregulation of endocannabinoids in models of adipose and beta‐pancreatic cells and in obesity and hyperglycemia. J Clin Endocrinol Metab 91: 3171–3180 16684820 10.1210/jc.2005-2679

[emmm202216225-bib-0054] Matthews E , Brassington R , Kuntzer T , Jichi F , Manzur AY (2016) Corticosteroids for the treatment of Duchenne muscular dystrophy. Cochrane Database Syst Rev 2016: CD003725 27149418 10.1002/14651858.CD003725.pub4 PMC8580515

[emmm202216225-bib-0055] McMahon DK , Anderson PA , Nassar R , Bunting JB , Saba Z , Oakeley AE , Malouf NN (1994) C2C12 cells: biophysical, biochemical, and immunocytochemical properties. Am J Physiol 266: C1795–C1802 8023908 10.1152/ajpcell.1994.266.6.C1795

[emmm202216225-bib-0056] Morais LH , Schreiber HL , Mazmanian SK (2020) The gut microbiota‐brain axis in behaviour and brain disorders. Nat Rev Microbiol 19: 241–255 33093662 10.1038/s41579-020-00460-0

[emmm202216225-bib-0057] Morosetti R , Broccolini A , Sancricca C , Gliubizzi C , Gidaro T , Tonali PA , Ricci E , Mirabella M (2010) Increased aging in primary muscle cultures of sporadic inclusion‐body myositis. Neurobiol Aging 31: 1205–1214 18823681 10.1016/j.neurobiolaging.2008.08.011

[emmm202216225-bib-0058] Morya R , Salvachúa D , Thakur IS (2020) Burkholderia: an untapped but promising bacterial genus for the conversion of aromatic compounds. Trends Biotechnol 38: 963–975 32818444 10.1016/j.tibtech.2020.02.008

[emmm202216225-bib-0059] Mulè F , Amato A , Serio R (2010) Gastric emptying, small intestinal transit and fecal output in dystrophic (mdx) mice. J Physiol Sci 60: 75–79 19784719 10.1007/s12576-009-0060-8 PMC10717827

[emmm202216225-bib-0060] O'Brien J , Hayder H , Zayed Y , Peng C (2018) Overview of microRNA biogenesis, mechanisms of actions, and circulation. Front Endocrinol 9: 402 10.3389/fendo.2018.00402 PMC6085463 30123182

[emmm202216225-bib-0061] Ortega‐Hernández A , Martínez‐Martínez E , Gómez‐Gordo R , López‐Andrés N , Fernández‐Celis A , Gutiérrrez‐Miranda B , Nieto ML , Alarcón T , Alba C , Gómez‐Garre D *et al* (2020) The interaction between mitochondrial oxidative stress and gut microbiota in the cardiometabolic consequences in diet‐induced obese rats. Antioxidants 9: 640 32708095 10.3390/antiox9070640 PMC7402124

[emmm202216225-bib-0062] Pagano C , Pilon C , Calcagno A , Urbanet R , Rossato M , Milan G , Bianchi K , Rizzuto R , Bernante P , Federspil G *et al* (2007) The endogenous cannabinoid system stimulates glucose uptake in human fat cells via phosphatidylinositol 3‐kinase and calcium‐dependent mechanisms. J Clin Endocrinol Metab 92: 4810–4819 17785353 10.1210/jc.2007-0768

[emmm202216225-bib-0063] Pane M , Vasta I , Messina S , Sorleti D , Aloysius A , Sciarra F , Mangiola F , Kinali M , Ricci E , Mercuri E (2006) Feeding problems and weight gain in Duchenne muscular dystrophy. Eur J Paediatr Neurol 10: 231–236 17045498 10.1016/j.ejpn.2006.08.008

[emmm202216225-bib-0064] Parada Venegas D , De la Fuente MK , Landskron G , González MJ , Quera R , Dijkstra G , Harmsen HJM , Faber KN , Hermoso MA (2019) Short chain fatty acids (SCFAs)‐mediated gut epithelial and immune regulation and its relevance for inflammatory bowel diseases. Front Immunol 10: 277 30915065 10.3389/fimmu.2019.00277 PMC6421268

[emmm202216225-bib-0065] Park C , Lee H , Hong S , Molagoda IMN , Jeong J‐W , Jin C‐Y , Kim G‐Y , Choi SH , Hong SH , Choi YH (2021) Inhibition of lipopolysaccharide‐induced inflammatory and oxidative responses by trans‐cinnamaldehyde in C2C12 myoblasts. Int J Med Sci 18: 2480–2492 34104079 10.7150/ijms.59169 PMC8176176

[emmm202216225-bib-0066] Paulson JN , Stine OC , Bravo HC , Pop M (2013) Differential abundance analysis for microbial marker‐gene surveys. Nat Methods 10: 1200–1202 24076764 10.1038/nmeth.2658 PMC4010126

[emmm202216225-bib-0067] Péladeau C , Adam NJ , Jasmin BJ (2018) Celecoxib treatment improves muscle function in mdx mice and increases Utrophin A expression. FASEB J 32: 5090–5103 29723037 10.1096/fj.201800081R

[emmm202216225-bib-0068] Prokopidis K , Chambers E , Ni Lochlainn M , Witard OC (2021) Mechanisms linking the gut‐muscle Axis with muscle protein metabolism and anabolic resistance: implications for older adults at risk of sarcopenia. Front Physiol 12: 770455 34764887 10.3389/fphys.2021.770455 PMC8576575

[emmm202216225-bib-0069] Quast C , Pruesse E , Yilmaz P , Gerken J , Schweer T , Yarza P , Peplies J , Glöckner FO (2013) The SILVA ribosomal RNA gene database project: improved data processing and web‐based tools. Nucleic Acids Res 41: D590–D596 23193283 10.1093/nar/gks1219 PMC3531112

[emmm202216225-bib-0070] Radley‐Crabb HG , Fiorotto ML , Grounds MD (2011) The different impact of a high fat diet on dystrophic mdx and control C57Bl/10 mice. PLoS Curr 3: RRN1276 22094293 10.1371/currents.RRN1276 PMC3217191

[emmm202216225-bib-0071] Richards JL , Yap YA , McLeod KH , Mackay CR , Mariño E (2016) Dietary metabolites and the gut microbiota: an alternative approach to control inflammatory and autoimmune diseases. Clin Transl Immunol 5: e82 10.1038/cti.2016.29 PMC4910123 27350881

[emmm202216225-bib-0072] Rojas‐Morales P , Tapia E , Pedraza‐Chaverri J (2016) β‐Hydroxybutyrate: a signaling metabolite in starvation response? Cell Signal 28: 917–923 27083590 10.1016/j.cellsig.2016.04.005

[emmm202216225-bib-0073] Runwal G , Stamatakou E , Siddiqi FH , Puri C , Zhu Y , Rubinsztein DC (2019) LC3‐positive structures are prominent in autophagy‐deficient cells. Sci Rep 9: 10147 31300716 10.1038/s41598-019-46657-z PMC6625982

[emmm202216225-bib-0074] Russell JT , Roesch LFW , Ördberg M , Ilonen J , Atkinson MA , Schatz DA , Triplett EW , Ludvigsson J (2019) Genetic risk for autoimmunity is associated with distinct changes in the human gut microbiome. Nat Commun 10: 3621 31399563 10.1038/s41467-019-11460-x PMC6689114

[emmm202216225-bib-0075] Sandri M , Coletto L , Grumati P , Bonaldo P (2013) Misregulation of autophagy and protein degradation systems in myopathies and muscular dystrophies. J Cell Sci 126: 5325–5333 24293330 10.1242/jcs.114041

[emmm202216225-bib-0076] Schwab M , Reynders V , Ulrich S , Zahn N , Stein J , Schröder O (2006) PPARgamma is a key target of butyrate‐induced caspase‐3 activation in the colorectal cancer cell line Caco‐2. Apoptosis 11: 1801–1811 16927016 10.1007/s10495-006-9788-2

[emmm202216225-bib-0078] Sheikh O , Yokota T (2020) Advances in genetic characterization and genotype‐phenotype correlation of Duchenne and Becker muscular dystrophy in the personalized medicine era. J Pers Med 10: 111 32899151 10.3390/jpm10030111 PMC7565713

[emmm202216225-bib-0079] Singh J , Verma NK , Kansagra SM , Kate BN , Dey CS (2007) Altered PPARγ expression inhibits myogenic differentiation in C2C12 skeletal muscle cells. Mol Cell Biochem 294: 163–171 16838108 10.1007/s11010-006-9256-x

[emmm202216225-bib-0080] Sun M , Wu W , Liu Z , Cong Y (2017) Microbiota metabolite short chain fatty acids, GPCR, and inflammatory bowel diseases. J Gastroenterol 52: 1–8 27448578 10.1007/s00535-016-1242-9 PMC5215992

[emmm202216225-bib-0081] Tang Y , Chen Y , Jiang H , Nie D (2011) Short‐chain fatty acids induced autophagy serves as an adaptive strategy for retarding mitochondria‐mediated apoptotic cell death. Cell Death Differ 18: 602–618 20930850 10.1038/cdd.2010.117 PMC3020988

[emmm202216225-bib-0082] Turcotte C , Chouinard F , Lefebvre JS , Flamand N (2015) Regulation of inflammation by cannabinoids, the endocannabinoids 2‐arachidonoyl‐glycerol and arachidonoyl‐ethanolamide, and their metabolites. J Leukoc Biol 97: 1049–1070 25877930 10.1189/jlb.3RU0115-021R

[emmm202216225-bib-0083] de Vos WM , Tilg H , Van Hul M , Cani PD (2022) Gut microbiome and health: mechanistic insights. Gut 71: 1020–1032 35105664 10.1136/gutjnl-2021-326789 PMC8995832

[emmm202216225-bib-0084] Walsh ME , Bhattacharya A , Sataranatarajan K , Qaisar R , Sloane L , Rahman MM , Kinter M , Van Remmen H (2015) The histone deacetylase inhibitor butyrate improves metabolism and reduces muscle atrophy during aging. Aging Cell 14: 957–970 26290460 10.1111/acel.12387 PMC4693467

[emmm202216225-bib-0085] Wishart DS , Feunang YD , Marcu A , Guo AC , Liang K , Vázquez‐Fresno R , Sajed T , Johnson D , Li C , Karu N *et al* (2018) HMDB 4.0: the human metabolome database for 2018. Nucleic Acids Res 46: D608–D617 29140435 10.1093/nar/gkx1089 PMC5753273

[emmm202216225-bib-0086] van de Wouw M , Boehme M , Lyte JM , Wiley N , Strain C , O'Sullivan O , Clarke G , Stanton C , Dinan TG , Cryan JF (2018) Short‐chain fatty acids: microbial metabolites that alleviate stress‐induced brain–gut axis alterations. J Physiol 596: 4923–4944 30066368 10.1113/JP276431 PMC6187046

