# Supplementary material for: Targeting gut dysbiosis against inflammation and impaired autophagy in Duchenne muscular dystrophy
Source: EMBO Mol Med. 2023 Jan 3;15(3):e16225. doi: 10.15252/emmm.202216225 (PMC9994484; doi:10.15252/emmm.202216225)
Supplement: Supplementary file 1 — Expanded View Figures PDF [file EMMM-15-e16225-s004.pdf]

Expanded View Figures

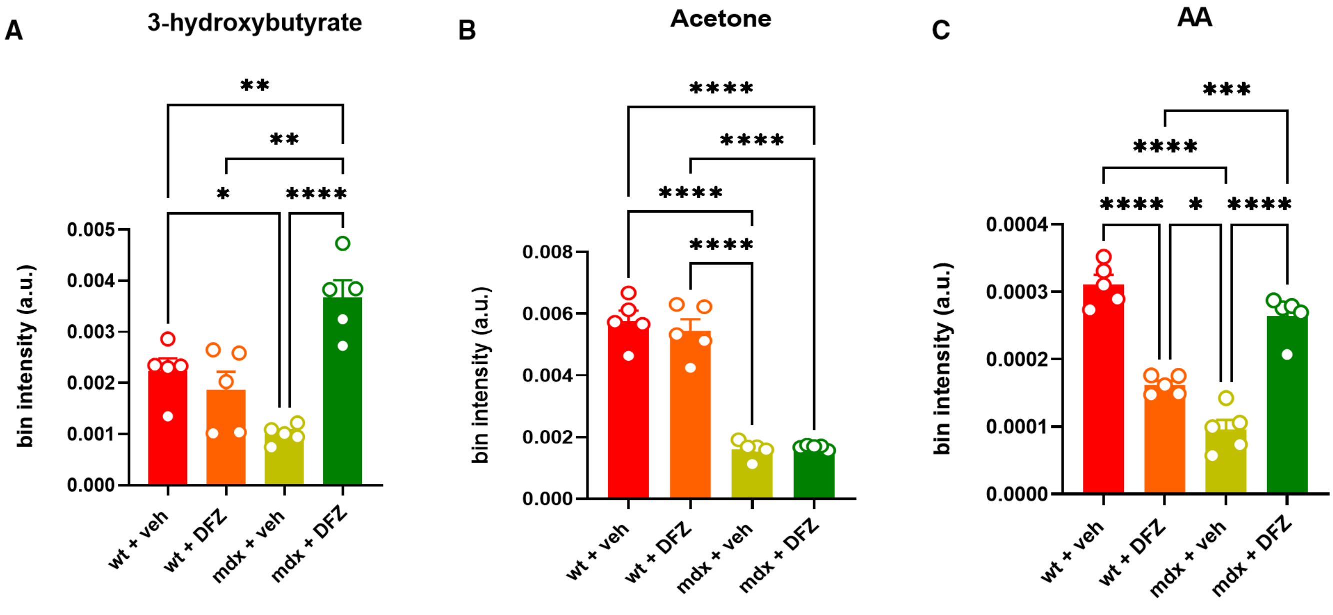

Figure EV1. Measurement of KBs in plasma samples of wt and mdx mice treated with or without DFZ.

A–C Bar chart with individual points showing the levels of the indicated metabolites detected in the plasma of wt and mdx mice treated ± DFZ. Data are expressed as bin intensity (a. u., arbitrary unit).

Data Information: Each bar is the mean ± S.E.M. of 5 independent biological determinations. ****$P \leq 0.0001$; ***$P \leq 0.0003$; **$P < 0.01$; *$P \leq 0.05$ vs. the indicated experimental group calculated using ANOVA.

 © 2023 The Authors

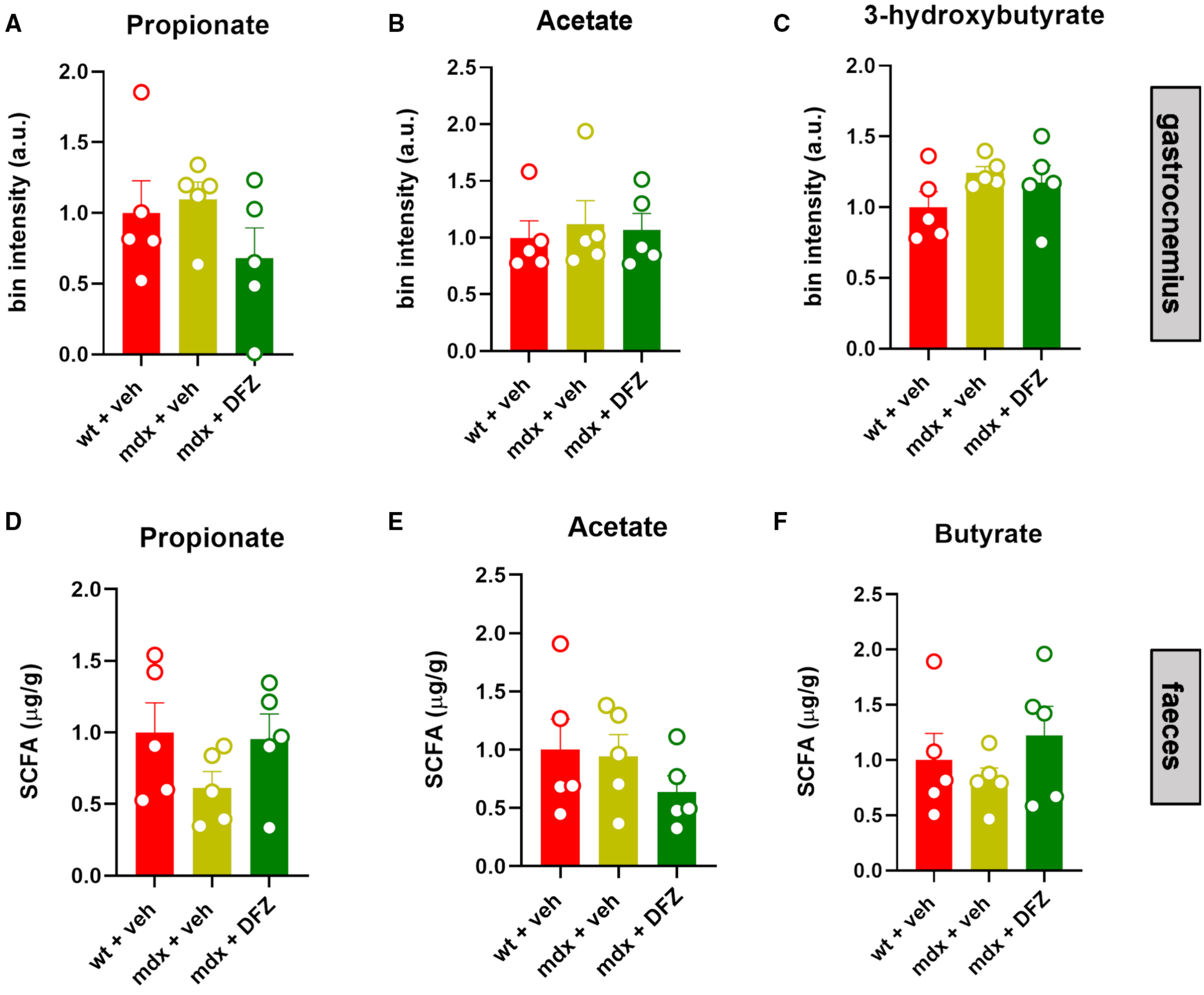

Figure EV2. Measurement of SCFAs and KBs in the gastrocnemius and fecal samples of wt and mdx mice treated with or without NaB or DFZ.

A–F Bar chart with individual points showing the levels of the indicated metabolites detected in the gastrocnemius and/or fecal samples of wt and mdx mice treated ± DFZ. Data are expressed as bin intensity (a. u., arbitrary unit).

Data Information: Each bar is the mean ± S.E.M. from 5 independent biological samples.

© 2023 The Authors

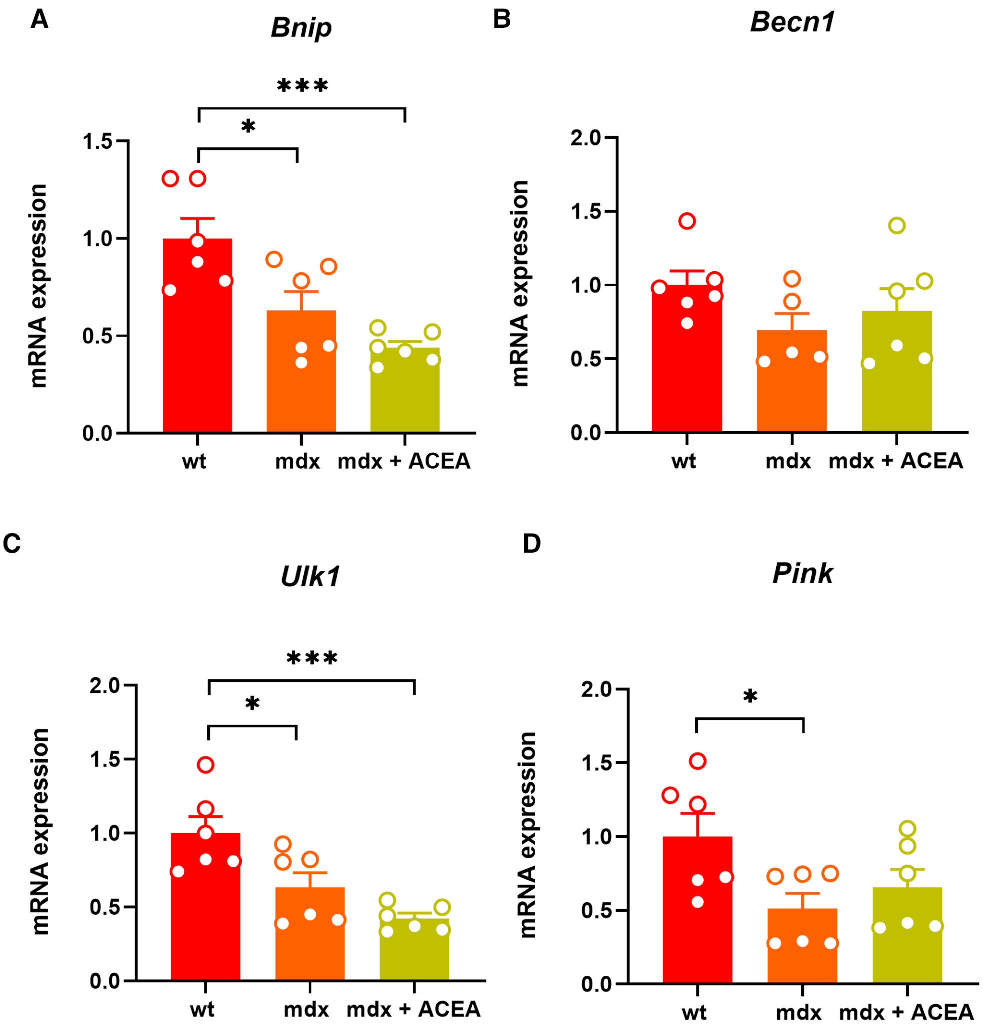

Figure EV3. Effect of ACEA on the expression of autophagy-related genes in mdx mice.

A–D Bar charts with individual points showing the mRNA expression levels of *Bnip*, *Becn1*, *Ulk1*, and *Pink* measured in control and mdx mice treated with ACEA 2.5 mg/kg.

Data Information: Each bar is the mean ± S.E.M. from 6 independent biological samples. ***$P \leq 0.0003$; *$P \leq 0.05$ vs. the indicated experimental group calculated using ANOVA.

© 2023 The Authors
