# Supplementary material for: Targeting gut dysbiosis against inflammation and impaired autophagy in Duchenne muscular dystrophy
Source: EMBO Mol Med. 2023 Jan 3;15(3):e16225. doi: 10.15252/emmm.202216225 (PMC9994484; doi:10.15252/emmm.202216225)

**Table EV1: GPR41, GPR43, GPR109A and PPARγ gene expression in C2C12 cells and gastrocnemius of control and mdx mice treated with DFZ or not.**


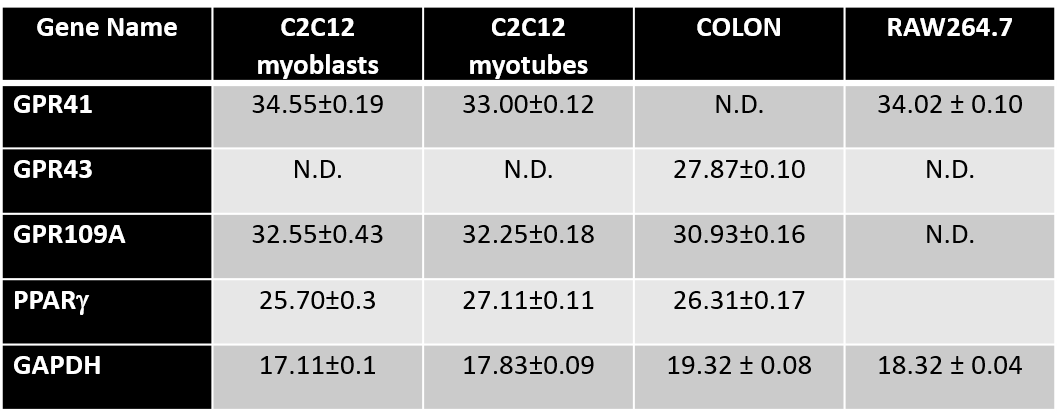

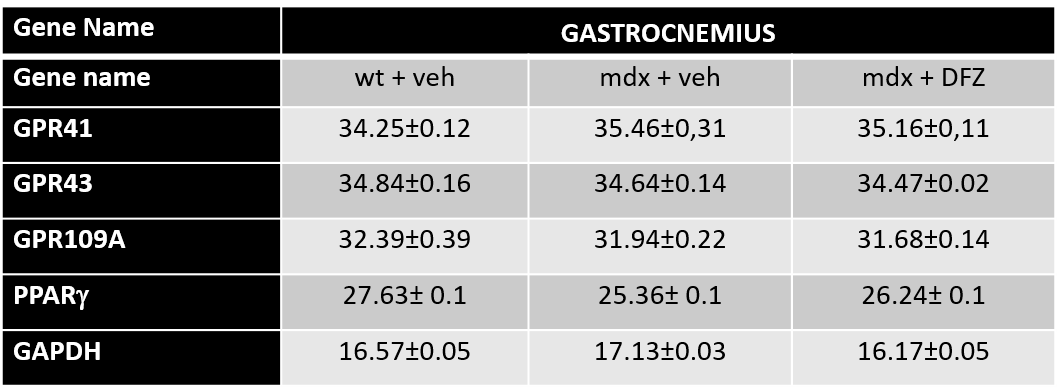

Supplement: Supplementary file 2 — Table EV1 [file EMMM-15-e16225-s003.docx]
