# Supplementary material for: Targeting gut dysbiosis against inflammation and impaired autophagy in Duchenne muscular dystrophy
Source: EMBO Mol Med. 2023 Jan 3;15(3):e16225. doi: 10.15252/emmm.202216225 (PMC9994484; doi:10.15252/emmm.202216225)
Supplement: Supplementary file 3 — Table EV2 [file EMMM-15-e16225-s001.doc]

**Table EV2: Clinical indices of DMD patients**

| **Patient:** | **Sex:** | **Age:** | **Biopsy diagnosis:** |
| --- | --- | --- | --- |
| **Donor 1 (D1)** | Male | 7 years 1 month | Exons 50-55 Deletion |
| **Donor 2 (D2)** | Male | 4 years 10 months | Exon 50 Deletion |
| **Donor 3 (D3)** | Male | 6 years 6 months | Exon 45 Deletion |
| **Donor 4 (D5)** | Male | 3 years 11 months | Exon 25 Stop mutation |
| **Donor 6 (D6)** | Male | 4 years 9 months | Exons 19-24 Deletion |
