# Supplementary figures and images for: Targeting gut dysbiosis against inflammation and impaired autophagy in Duchenne muscular dystrophy
Source: EMBO Mol Med. 2023 Jan 3;15(3):e16225. doi: 10.15252/emmm.202216225 (PMC9994484; doi:10.15252/emmm.202216225)

**Table EV3: List of murine and human primer sequences**


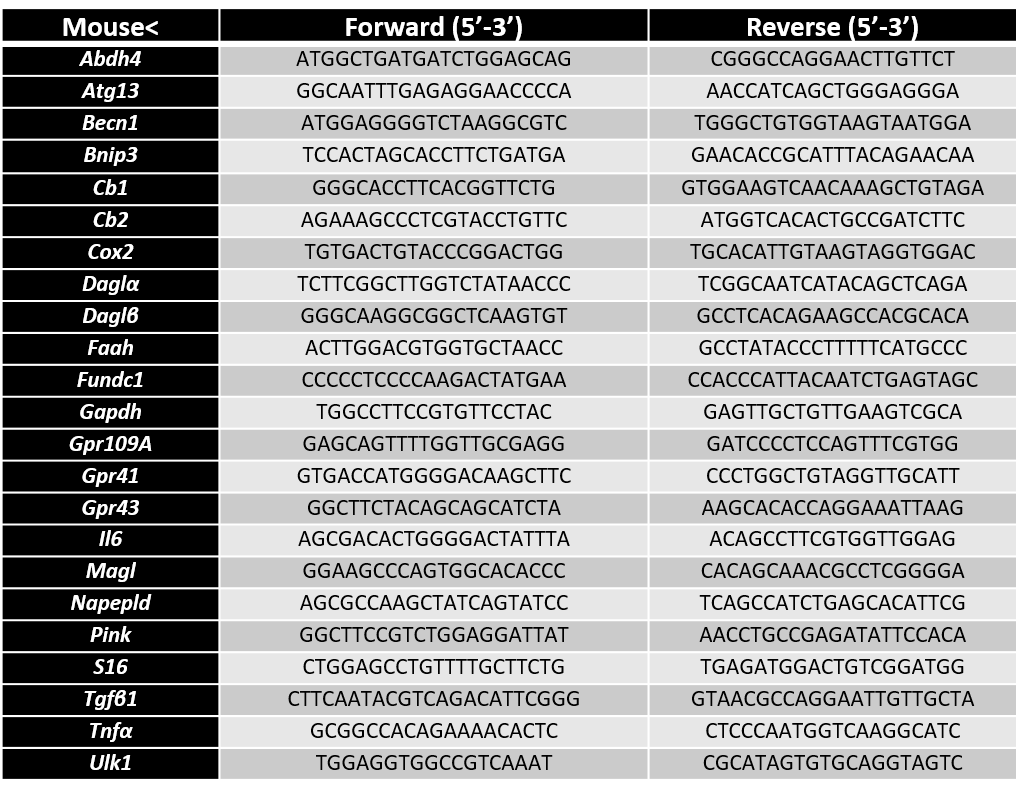

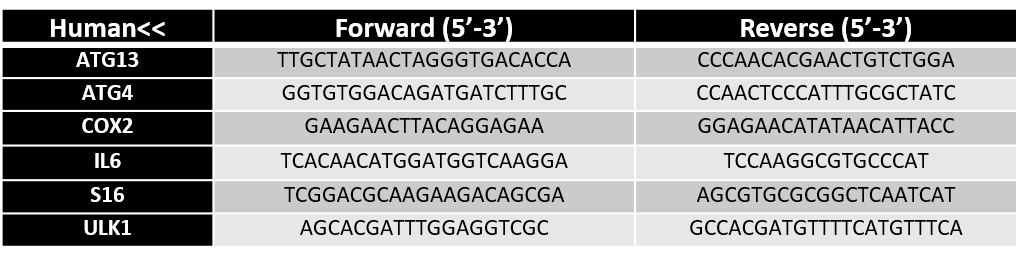

Supplement: Supplementary file 4 — Table EV3 [file EMMM-15-e16225-s005.doc]
