# Supplementary material for: Targeting gut dysbiosis against inflammation and impaired autophagy in Duchenne muscular dystrophy
Source: EMBO Mol Med. 2023 Jan 3;15(3):e16225. doi: 10.15252/emmm.202216225 (PMC9994484; doi:10.15252/emmm.202216225)
Supplement: Supplementary file 6 [file EMMM-15-e16225-s002.pdf]

EMBO Molecular Medicine

Targeting gut dysbiosis against inflammation and impaired autophagy in Duchenne muscular dystrophy

Hilal Kalkan, Ester Pagano, Debora Paris, Elisabetta Panza, Mariarosaria Cuozzo, Claudia Moriello, Fabiana Piscitelli, Armita Abolghasemi, Elisabetta Gazzerro, Cristoforo Silvestri, Raffaele Capasso, Andrea Motta, Roberto Russo, Vincenzo Di Marzo, and Fabio Iannotti
DOI: 10.15252/emmm.202216225

Corresponding authors: Fabio Iannotti (fabio.iannotti@icb.cnr.it) , Vincenzo Di Marzo (vincenzo.dimarzo@criucpq.ulaval.ca)

Review Timeline:

Submission Date:	26th Apr 22
Editorial Decision:	20th Jun 22
Revision Received:	21st Nov 22
Editorial Decision:	12th Dec 22
Revision Received:	13th Dec 22
Accepted:	14th Dec 22

Editor: Jingyi Hou

Transaction Report:

(Note: With the exception of the correction of typographical or spelling errors that could be a source of ambiguity, letters and reports are not edited. Depending on transfer agreements, referee reports obtained elsewhere may or may not be included in this compilation. Referee reports are anonymous unless the Referee chooses to sign their reports.)

21st Jun 2022

Dear Dr. Iannotti,

Thank you for submitting your work to EMBO Molecular Medicine. First of all, I would like to apologize for the slow process. We have now heard back from two of the three referees who agreed to evaluate your manuscript. Unfortunately, after a series of reminders, we did not manage to obtain a report from Referee #3. In the interest of time, I prefer to make a decision now rather than further delay the process. If we receive the comments from Referee #3, we will send them to you, and you can address the issues raised by Referee #3 together with those raised by the other two referees. You will see from the comments below that the referees think the presented findings are interesting and novel. They raise, however, several important points, which should be convincingly addressed in a revision of this work.

I think the referees' recommendations are rather clear, and there is no need for me to reiterate their comments. Importantly, Referee #2 pointed out that several critical control experiments are missing, which must be carefully addressed.

All other issues raised by the reviewers need to be addressed as well. We would welcome the submission of a revised version within three months for further consideration. Please note that EMBO Molecular Medicine strongly supports a single round of revision. As acceptance or rejection of the manuscript will depend on another round of review, your responses should be as complete as possible.

EMBO Molecular Medicine has a "scooping protection" policy, whereby similar findings that are published by others during review or revision are not a criterion for rejection. Should you decide to submit a revised version, I do ask that you get in touch after three months if you have not completed it to update us on the status.

We are aware that many laboratories cannot function at full efficiency during the current COVID-19/SARS-CoV-2 pandemic and have therefore extended our "scooping protection policy" to cover the period required for a full revision to address the experimental issues. Please let me know should you need additional time and also if you see a paper with related content published elsewhere.

Please read below for important editorial formatting and consult our author's guidelines for proper formatting of your revised article for EMBO Molecular Medicine.

I look forward to receiving your revised manuscript.

Use this link to login to the manuscript system and submit your revision: https://embomolmed.msubmit.net/cgi-bin/main.plex

Kind regards,
Jingyi

Jingyi Hou
Editor
EMBO Molecular Medicine

***** Reviewer's comments *****

Referee #1 (Comments on Novelty/Model System for Author):

This study follows a logical progression which builds upon our understanding of DMD inflammatory and autophagy dysregulation which has been previously reported. Novelty and impact are somewhat impacted because previous studies have shown that activation of AKT and mTOR pathways (downstream of CB1 receptor activation) suppress autophagy.

Referee #1 (Remarks for Author):

This study by Kalkan et al. is well written and with well thought out experiments and data presentation. The work pushes our understanding of the potential impact altered gut microbiota may be having on reducing autophagy in the skeletal muscle of dystrophin deficient mice and for Duchenne muscular dystrophy patients. Overall I believe the work is close to being ready for publication. I have included some thoughts below which should be considered.

1. A more comprehensive overview of the known biological roles sodium butyrate plays should be included to help readers. Sodium Butyrate is a known HDAC inhibitor but has poor pharmaceutical potential due to first pass hepatic clearance. The finding of normal levels of plasma Butyrate by GC mass is somewhat contradictory to the overall story.

2. Referencing of previous work on autophagy in DMD should be expanded. For example, overactivation of p-AKT and mTOR, both potential downstream targets of CB1 and PPAR, have been previously reported to reduce autophagy in DMD. This seems like a logical reference and work that you have expanded upon.

Notes:

1. Iannotti reference to C2C12 cell characterization on page 5 is inappropriate. C2C12 cells have been used/referenced by many other groups prior to 2010.

2. Figure 6B had altered bar graph colors from others.

Referee #2 (Comments on Novelty/Model System for Author):

The manuscript describes a potential intervention therapy to attenuate the symptoms of the accelerating disease in DMD. The data are clear and very interesting. There are controls missing but if the authors can complement I am in strong favor of rapid publication.

Referee #2 (Remarks for Author):

In this paper, entitled "Targeting gut dysbiosis against inflammation and impaired autophagy in Duchenne muscular", by Hilal Kalkan et al, the authors explore the effects of altering the observed gut dysbiosis and its relevance to the mouse model of Duchenne Muscular Dystrophy (DMD).
The authors report evidence that faecal microbiota together with circulating levels of the metabolites
acetate, propionate, and butyrate are altered in the "mdx" mouse model.
Introducing sodium butyrate (NaB) in "mdx" transgenic mice rescues muscle strength and autophagy. In addition, the butyrate also prevented inflammation associated with an increased endocannabinoid signaling at CB1 receptors, similar to deflazacort (DFZ), the standard palliative care for DMD. This phenomenon is also corroborated in cell line experiments. Specifically, Sodium butyrate reduces inflammation and prevents dysregulation of microRNA targeting the endocannabinoid CB1 receptor gene in a manner depending on the activation of GPR109A and PPARγ receptors. The manuscript provides interesting insights into the pathogenesis of DMD.
General comment.
The manuscript is well written, and the figures overall support the interpretation of their findings. The problem is what the authors do not show and the choice of the timepoint of recording of impact on SCFA treatment. That SCFA has beneficial effects on skeletal muscle growth have been reported and should be included (Lahiri et al 2019)
Specifically
Figure 2. The data presented in figure 2 are convincing but why did the authors not include the DFZ in WT mice? Please include. Moreover, the lack of including treatment of interest in the WT recipient mice is missing in many figures and needs to be corrected when appropriate.
In addition, while minor, SCFA has been shown to reduce the production of HPA-mediated increase in cortisol levels (Neuropsychopharmacology (2020) 45:2257-2266; https://doi.org/10.1038/s41386-020-0732-xcortisol). The authors may want to comment on this.
Figure 3. The functional data are clear. But the authors must include the NaB and DFZ on WT mice as an important control. Please also include proper histopathology and relevant immunohistochemistry to demonstrate muscle tissue effects before and after treatment. At present, it is not clear if the functional effects observed are direct in the skeletal muscle or an indirect effect by improved central coordination of peripheral muscle function.

Figure 4. Inflammation and Autophagy recording. Again, overall convincing data. But the time point of recording is somewhat surprising as the inflammation is assumed to peak earlier (around two months, see (see 2020) 10:14070 | https://doi.org/10.1038/s41598-020-70987-y). At three months of age, inflammation is declining. Can the authors provide data from two Months old mice? In addition, the authors must include the monitoring TGFb1 and mRNA and protein level.
Figure 5. Again, convincing data but lacking WT mice exposed to DFZ and NaB. Please include as a control
Figure 9. Interesting preliminary finding but sample size too small. In addition, no muscle tissue from donor patients is presented. The authors must expand the sample size. Alternatively, consider taking out from the study.

Referee #1 (Comments on Novelty/Model System for Author): "This study follows a logical progression which builds upon our understanding of DMD inflammatory and autophagy dysregulation which has been previously reported. Novelty and impact are somewhat impacted because previous studies have shown that activation of AKT and mTOR pathways (downstream of CB1 receptor activation) suppress autophagy". **We thank the reviewer for the positive comment. We appreciate his/her suggestion to remark on the concept that AKT and mTOR pathways are linked to CB1 receptor signalling (see lines 401-403; page 8).**

Referee #1 (Remarks for Author):

"This study by Kalkan et al. is well written and with well thought out experiments and data presentation. The work pushes our understanding of the potential impact altered gut microbiota may be having on reducing autophagy in the skeletal muscle of dystrophin deficient mice and for Duchenne muscular dystrophy patients. Overall I believe the work is close to being ready for publication. I have included some thoughts below which should be considered". **We thank the reviewer once again for the positive comment.**

1. A more comprehensive overview of the known biological roles sodium butyrate plays should be included to help readers. Sodium Butyrate is a known HDAC inhibitor but has poor pharmaceutical potential due to first pass hepatic clearance. The finding of normal levels of plasma Butyrate by GC mass is somewhat contradictory to the overall story. **We understand the concern raised by the reviewer. However, there is a consistent number of studies demonstrating and supporting the future development of probiotics and next-generation probiotics (NGS) using butyrate-producing bacterial species to treat human diseases. For instance, a randomized clinical study by Zhao et al. demonstrated that supplementation of a mix of dietary fibres to individuals with type 2 diabetes - T2D- improved glycemic parameters, accompanied by an increased abundance of acetate- and butyrate-producing bacteria and increased faecal levels of acetate and butyrate (Zhao *et al*, 2018). In another study, performed also in individuals with T2D, a mix of bacteria species producing butyrate together with inulin increased butyrate levels and improved oral glucose tolerance and glycated haemoglobin levels (Perraudeau *et al*, 2020). Again other investigators reported the therapeutic value of bacteria-producing butyrate in patients with Crohn's and/or inflammatory bowel diseases (Geirnaert *et al*, 2017; Parada Venegas *et al*, 2019). Similar effects were also seen in patients with obesity-associated metabolic disorders (XU *et al*, 2022). Of course, we are not proposing to use butyrate per se as a treatment, but we believe that our data may lead to proposing butyrate-producing strategies, such as the enhancement of the relative abundance of butyrate-producing**

commensal bacteria by employing suitable prebiotics, or the use of butyrate-producing probiotics, as a potential co-treatment for DMD.

We also agree with the reviewer about the apparent discrepancy in butyrate levels we found between control and mdx mice. However, we do see a trend for a decrease in butyrate levels in the faeces of mdx mice (Fig. EV2) and, perhaps most importantly, we find, butyrate in the plasma, a significant decrease in 3-hydroxy-butyrate (Fig, EV1), which can be produced from butyrate after first-pass hepatic clearance, possibly suggesting an overall reduced production of butyrate by gut microbiota. Importantly both the trend in the faeces and the decrease of 3-hydroxy-butyrate in the plasma were reverted by DFZ. It is also worth mentioning that also in other studies it is reported that under specific pathological circumstances, the butyrate concentration in plasma does not change either after exogenous supplementation, although the whole body turnover is significantly modified (Zhao *et al*, 2018; Perry *et al*, 2016). These authors, in particular, state that circulating or intestinal butyrate content does not always reflect its endogenous production and turnover. However, future studies to define butyrate production and turnover as well as the influence of environmental factors in DMD are certainly needed.

2. Referencing of previous work on autophagy in DMD should be expanded. For example, overactivation of p-AKT and mTOR, both potential downstream targets of CB1 and PPAR, have been previously reported to reduce autophagy in DMD. This seems like a logical reference and work that you have expanded upon. **We thank the reviewer for the constructive suggestion, and we have integrated the text of the discussion to mention these relevant findings (see lines 401-403; page 8).**

Notes:

1. Iannotti reference to C2C12 cell characterization on page 5 is inappropriate. C2C12 cells have been used/referenced by many other groups prior to 2010. **According to this request, we changed the reference (line 570, page 11).**

2. Figure 6B had altered bar graph colors from others. We apologize for the mistake. **The figure has been revised.**

Referee #2 (Comments on Novelty/Model System for Author):

"The manuscript describes a potential intervention therapy to attenuate the symptoms of the accelerating disease in DMD. The data are clear and very interesting. There are controls missing but if the authors can complement I am in strong favor of rapid publication". **We thank the reviewer for the positive comment and constructive suggestions.**

Referee #2 (Remarks for Author):

General comment.

The manuscript is well written, and the figures overall support the interpretation of their findings. The problem is what the authors do not show and the choice of the timepoint of recording of impact on SCFA treatment. That SCFA has beneficial effects on skeletal muscle growth have been reported and should be included (Lahiri et al 2019). **We thank the reviewer for the positive comment and constructive suggestions she/he made throughout the manuscript. According to her/his specific request, the study of Lahiri et al. has been emphasized (line 73, page 2).**

Specifically

1. Figure 2. The data presented in figure 2 are convincing but why did the authors not include the DFZ in WT mice? Please include. **According to this request, the experimental group wt+DFZ has been included in Fig. 2.**

2. Moreover, the lack of including treatment of interest in the WT recipient mice is missing in many figures and needs to be corrected when appropriate. **According to this request, the effect of DFZ and NaB in healthy mice is now shown in Figures 3, 4 and 5.**

3. In addition, while minor, SCFA has been shown to reduce the production of HPA-mediated increase in cortisol levels (Neuropsychopharmacology (2020) 45:2257-2266; https://doi.org/10.1038/s41386-020-0732-xcortisol). The authors may want to comment on this. **According to this request, we mentioned the study of Dalile et al. as well as others in the discussion (lines 377-386, page 8).**

4. Figure 3. The functional data are clear. But the authors must include the NaB and DFZ on WT mice as an important control. **According to this request, we included the missing control groups in Fig. 3**

5. Please also include proper histopathology and relevant immunohistochemistry to demonstrate muscle tissue effects before and after treatment. **Regrettably, we are not able to provide these results. During the execution of these experiments, there was an unexpected breakdown of the lasers of the confocal microscope which seemed like an easily solvable problem but it was not. After that, we did not have time to ask other collaborators to carry out the experiments in the allotted time. We deeply apologize for the inconvenience. However, we would like to draw the attention of this reviewer to the fact that in our previous study, we did produce histopathological and morphological studies in support of the fact that pharmacological and genetic blockade of CB1 receptors can indeed ameliorate muscle structure in mdx mice (Iannotti *et al*, 2018). Since we show in our present study that also butyrate produces its beneficial effects by counteracting CB1 receptor activity, we have no reason to believe that, had we had the possibility of performing these experiments, we would have found also morphological evidence of the protective action of butyrate.**

6. At present, it is not clear if the functional effects observed are direct in the skeletal muscle or an indirect effect by improved central coordination of peripheral muscle function. **Our study demonstrates that daily supplementation with NaB to mdx mice changes the expression of key genes regulating inflammation and autophagy as well as the endocannabinoid system activity in skeletal muscles. Moreover, we also provide mechanistic insights into the effects of NaB obtained in LPS-stimulated C2C12 myoblasts. Therefore although it is clear that NaB exerts a beneficial effect on muscle tissues, we cannot exclude its action in the central nervous system, particularly as we could not detect changes in butyrate levels in the skeletal muscle of mdx mice. However, to the best of our knowledge, there is no evidence that NaB used at 100 mg/Kg could interfere with central coordination.**

In the next years, our research activity will be focused on exploring the gut microbiota-brain axis in DMD. During the revision of this manuscript, F.A.I. received a grant from Duchenne Parent Project NL to carry out the study. Thus, we will keep in mind this precious comment and hope to provide novel insight into this subject.

7. Figure 4. Inflammation and Autophagy recording. Again, overall convincing data. But the time point of recording is somewhat surprising as the inflammation is assumed to peak earlier (around two months, see (see 2020) 10:14070 | https://doi.org/10.1038/s41598-020-70987-y). At three months of age, inflammation is declining. Can the authors provide data from two Months old mice? **We understand and agree with the point raised by the reviewer. However, it is reported that in mdx mice older than eight weeks the inflammation is still persistent with levels of pro-inflammatory factors not diminished or in any case significantly high (Lagrota-Candido *et al*, 2002). However, to fulfil this request, we were able to produce preliminary data (n=3) demonstrating that treatment with sodium butyrate significantly reduces the expression of inflammation markers in 8-week-old mdx mice. For now, we have not inserted these data in the manuscript, but are ready to do so on request or to mention them as data not shown.**

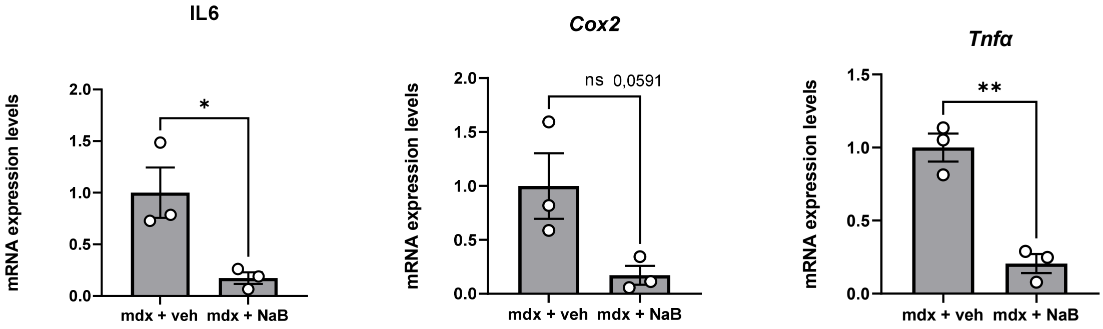

8. In addition, the authors must include the monitoring TGFb1 and mRNA and protein level. **To fulfil this request, we measured TGFb1 levels measured in the skeletal muscle of mice subject to our experimental conditions. mRNA levels of TGFb1 are shown below. Regarding the western blot analysis, we performed the experiments using a commercially available antibody that we purchased from Thermo Fisher (MA5-15065). However, using this antibody we could not detect any signal for TGF beta in our samples (see the representative blot below). We are inclined to believe that the quality of the antibody is most likely poor, given the presence of non-specific bands. The other option is that in most tissues of mdx mice the fibrotic process peaks later than 21-22 weeks as reported by many other investigators (Morrison *et al*, 2000; Au *et al*, 2011; Lagrota-Candido *et al*, 2002). For now, we have not inserted these data in the manuscript, but are ready to do so on request or to mention them as data not shown.**

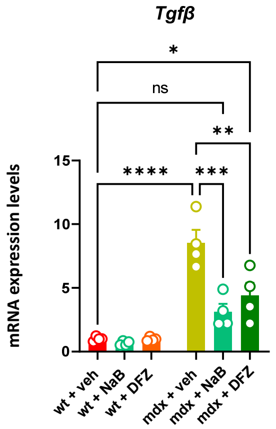

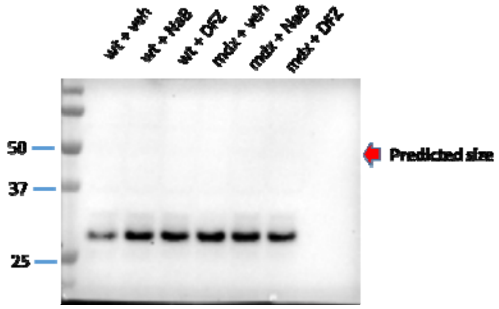

9. Figure 5. Again, convincing data but lacking WT mice exposed to DFZ and NaB. Please include as a control. **According to this request, we included the missing experimental groups in Fig. 5**

10. Figure 9. Interesting preliminary finding but sample size too small. In addition, no muscle tissue from donor patients is presented. The authors must expand the sample size. Alternatively, consider taking out from the study. **Also according to this request, we have implemented the analysis using RNA samples isolated from primary skeletal muscle cells obtained from other two DMD donors.**

1st Revision - Editorial Decision 12th Dec 2022

12th Dec 2022

Dear Dr. Iannotti,

Thank you for the submission of your revised manuscript to EMBO Molecular Medicine. We have now received the enclosed report from the referee, who agreed to re-assess it. As you will see, the referee is now overall supportive, and I am pleased to inform you that we will be able to accept your manuscript pending the following amendments:

1. Remove the yellow color font.

2. We updated our journal's competing interests policy in January 2022 and request authors to consider both actual and perceived competing interests. Please review the policy https://www.embopress.org/competing-interests and update your competing interests if necessary.
Please use the heading "Disclosure statement and competing interests".

3. Remove the Author contribution section from the manuscript text.

4. Please provide individual production-quality figure files（for both main figures and EV figures） as .eps, .tif, .jpg (one file per figure). Figure legends should be removed from figures and should be in the manuscript.

5. Figure and table callouts- please fix the following issues:
-Callouts are missing for Fig 4H and I; Fig 5B; Fig 6D; Table EV2;
-Figure EV3 should not be called out before Figure EV2.
-There are callouts for Fig 9E-J but no such panels.
-There is a callout for Suppl Table 3; should this be Table EV3?

6. Data availability
- primary datasets produced in this study (sequencing data) need to be deposited in an appropriate public database (such as GEO etc). The accession numbers and database should be listed in a formal "Data Availability" section that follows the model below (see also https://www.embopress.org/page/journal/17574684/authorguide#dataavailability).
Data availability

The datasets (and computer code) produced in this study are available in the following databases:

- RNA-Seq data: Gene Expression Omnibus GSE46843 (https://www.ncbi.nlm.nih.gov/geo/query/acc.cgi?acc=GSE46843)
- [data type]: [name of the resource] [accession number/identifier/doi] ([URL oridentifiers.org/DATABASE:ACCESSION])

7. Please incorporate The Paper Explained into the main manuscript text.

8. I have slightly shortened and modified the synopsis text(see attached). Please let me know if it is fine as is or if you would like to introduce further modifications.

9. Our data editors have seen the manuscript, and they have made some comments and suggestions that need to be addressed (see attached). Please send back a revised version (in track change mode), as we will need to go through the changes.

10. As part of the EMBO Publications transparent editorial process initiative (see our Editorial at http://embomolmed.embopress.org/content/2/9/329), EMBO Molecular Medicine will publish online a Review Process File (RPF) to accompany accepted manuscripts.

In the event of acceptance, this file will be published in conjunction with your paper and will include the anonymous referee reports, your point-by-point response, and all pertinent correspondence relating to the manuscript. Let us know whether you disagree with this and if you want to remove or keep any figures from it prior to publication.

Please note that the Authors checklist will be published at the end of the RPF.

Please submit your revised manuscript by December 21st, and ideally as soon as possible.

Kind regards,
Jingyi

Jingyi Hou
Editor
EMBO Molecular Medicine

***** Reviewer's comments *****

Referee #2 (Comments on Novelty/Model System for Author):

The authors have largely responded well to my comments. Yet, there are parts missing, though they give a reasonable explanation
it's an interesting report that warrants publication in an area always looking for potential ways to improve the quality of life for patients with Duchenne muscle dystrophia

Referee #2 (Remarks for Author):

I have no further comments

The authors addressed the minor editorial issues.

14th Dec 2022

Dear Fabio,

I am pleased to inform you that your manuscript is accepted for publication and is now being sent to our publisher to be included in the next available issue of EMBO Molecular Medicine.

We would like to remind you that as part of the EMBO Publications transparent editorial process initiative, EMBO Molecular Medicine will publish a Review Process File online to accompany accepted manuscripts. If you do NOT want the file to be published or would like to exclude figures, please immediately inform the editorial office via e-mail.

Please read below for additional IMPORTANT information regarding your article, its publication and the production process.

Congratulations on your interesting work,

Best wishes,
Jingyi

Jingyi Hou
Editor
EMBO Molecular Medicine

Follow us on Twitter @EmboMolMed
Sign up for eTOCs at embopress.org/alertsfeeds

*** *** *** IMPORTANT INFORMATION *** *** ***

SPEED OF PUBLICATION
The journal aims for rapid publication of papers, using using the advance online publication "Early View" to expedite the process: A properly copy-edited and formatted version will be published as "Early View" after the proofs have been corrected. Please help the Editors and publisher avoid delays by providing e-mail address(es), telephone and fax numbers at which author(s) can be contacted.

Should you be planning a Press Release on your article, please get in contact with embomolmed@wiley.com as early as possible, in order to coordinate publication and release dates.

LICENSE AND PAYMENT:

All articles published in EMBO Molecular Medicine are fully open access: immediately and freely available to read, download and share.

EMBO Molecular Medicine charges an article processing charge (APC) to cover the publication costs. You, as the corresponding author for this manuscript, should have already received a quote with the article processing fee separately. Please let us know in case this quote has not been received.

Once your article is at Wiley for editorial production you will receive an email from Wiley's Author Services system, which will ask you to log in and will present you with the publication license form for completion. Within the same system the publication fee can be paid by credit card, an invoice, pro forma invoice or purchase order can be requested.

Payment of the publication charge and the signed Open Access Agreement form must be received before the article can be published online.

PROOFS

You will receive the proofs by e-mail approximately 2 weeks after all relevant files have been sent o our Production Office. Please return them within 48 hours and if there should be any problems, please contact the production office at embopressproduction@wiley.com.

Please inform us if there is likely to be any difficulty in reaching you at the above address at that time. Failure to meet our deadlines may result in a delay of publication.

All further communications concerning your paper proofs should quote reference number EMM-2022-16225-V3 and be directed to the production office at embopressproduction@wiley.com.

Thank you,

Jingyi Hou
Editor
EMBO Molecular Medicine

EMBO Press Author Checklist

Corresponding Author Name: IANNOTTI FABIO ARTURO	
Journal Submitted to: EMBO MOLECULAR MEDICINE	
Manuscript Number: EMM-2022-16225	

USEFUL LINKS FOR COMPLETING THIS FORM
The EMBO Journal - Author Guidelines
EMBO Reports - Author Guidelines
Molecular Systems Biology - Author Guidelines
EMBO Molecular Medicine - Author Guidelines

Reporting Checklist for Life Science Articles (updated January 2022)
This checklist is adapted from Materials Design Analysis Reporting (MDAR) Checklist for Authors. MDAR establishes a minimum set of requirements in transparent reporting in the life sciences (see Statement of Task: 10.31222/osf.io/9sm4x). Please follow the journal's guidelines in preparing your manuscript.
Please note that a copy of this checklist will be published alongside your article.

Abridged guidelines for figures
1. Data
The data shown in figures should satisfy the following conditions:
- the data were obtained and processed according to the field's best practice and are presented to reflect the results of the experiments in an accurate and unbiased manner.
- ideally, figure panels should include only measurements that are directly comparable to each other and obtained with the same assay.
- plots include clearly labeled error bars for independent experiments and sample sizes. Unless justified, error bars should not be shown for technical replicates.
- if n<5, the individual data points from each experiment should be plotted. Any statistical test employed should be justified.
- Source Data should be included to report the data underlying figures according to the guidelines set out in the authorship guidelines on Data Presentation.

2. Captions
Each figure caption should contain the following information, for each panel where they are relevant:
- a specification of the experimental system investigated (eg cell line, species name).
- the assay(s) and method(s) used to carry out the reported observations and measurements.
- an explicit mention of the biological and chemical entity(ies) that are being measured.
- an explicit mention of the biological and chemical entity(ies) that are altered/varied/perturbed in a controlled manner.
- the exact sample size (n) for each experimental group/condition, given as a number, not a range;
- a description of the sample collection allowing the reader to understand whether the samples represent technical or biological replicates (including how many animals, litters, cultures, etc.).
- a statement of how many times the experiment shown was independently replicated in the laboratory.
- definitions of statistical methods and measures:
  - common tests, such as t-test (please specify whether paired vs. unpaired), simple χ2 tests, Wilcoxon and Mann-Whitney tests, can be unambiguously identified by name only, but more complex techniques should be described in the methods section;
  - are tests one-sided or two-sided?
  - are there adjustments for multiple comparisons?
  - exact statistical test results, e.g., P values = x but not P values < x;
  - definition of 'center values' as median or average;
  - definition of error bars as s.d. or s.e.m.

Please complete ALL of the questions below.
Select "Not Applicable" only when the requested information is not relevant for your study.

Materials

Newly Created Materials	Information included in the manuscript?	In which section is the information available? (Reagents and Tools Table, Materials and Methods, Figures, Data Availability Section)
New materials and reagents need to be available; do any restrictions apply?	Not Applicable	

Antibodies	Information included in the manuscript?	In which section is the information available? (Reagents and Tools Table, Materials and Methods, Figures, Data Availability Section)
For **antibodies** provide the following information:<br>- Commercial antibodies: RRID (if possible) or supplier name, catalogue number and or/clone number<br>- Non-commercial: RRID or citation	Yes	Materials and Methods

DNA and RNA sequences	Information included in the manuscript?	In which section is the information available? (Reagents and Tools Table, Materials and Methods, Figures, Data Availability Section)
**Short novel DNA or RNA including primers, probes**: provide the sequences.	Not Applicable	

Cell materials	Information included in the manuscript?	In which section is the information available? (Reagents and Tools Table, Materials and Methods, Figures, Data Availability Section)
**Cell lines:** Provide species information, strain. Provide accession number in repository **OR** supplier name, catalog number, clone number, and/**OR** RRID.	Yes	Materials and Methods
**Primary cultures:** Provide species, strain, sex of origin, genetic modification status.	Yes	Materials and Methods
Report if the cell lines were recently **authenticated** (e.g., by STR profiling) and tested for mycoplasma contamination.	Not Applicable	

Experimental animals	Information included in the manuscript?	In which section is the information available? (Reagents and Tools Table, Materials and Methods, Figures, Data Availability Section)
**Laboratory animals or Model organisms:** Provide species, strain, sex, age, genetic modification status. Provide accession number in repository **OR** supplier name, catalog number, clone number, **OR** RRID.	Yes	Materials and Methods
**Animal observed in or captured from the field:** Provide species, sex, and age where possible.	Not Applicable	
Please detail **housing and husbandry conditions**.	Yes	Materials and Methods

Plants and microbes	Information included in the manuscript?	In which section is the information available? (Reagents and Tools Table, Materials and Methods, Figures, Data Availability Section)
**Plants:** provide species and strain, ecotype and cultivar where relevant, unique accession number if available, and source (including location for collected wild specimens).	Not Applicable	
**Microbes:** provide species and strain, unique accession number if available, and source.	Not Applicable	

Human research participants	Information included in the manuscript?	In which section is the information available? (Reagents and Tools Table, Materials and Methods, Figures, Data Availability Section)
If collected and within the bounds of privacy constraints report on age, sex and gender or ethnicity for all study participants.	Not Applicable	

Core facilities	Information included in the manuscript?	In which section is the information available? (Reagents and Tools Table, Materials and Methods, Figures, Data Availability Section)
If your work benefited from core facilities, was their service mentioned in the acknowledgments section?	Not Applicable	

Design

Study protocol	Information included in the manuscript?	In which section is the information available? (Reagents and Tools Table, Materials and Methods, Figures, Data Availability Section)
If study protocol has been **pre-registered, provide DOI in the manuscript**. For clinical trials, provide the trial registration number **OR** cite DOI.	Not Applicable	
Report the **clinical trial registration number** (at ClinicalTrials.gov or equivalent), where applicable.	Not Applicable	

Laboratory protocol	Information included in the manuscript?	In which section is the information available? (Reagents and Tools Table, Materials and Methods, Figures, Data Availability Section)
Provide DOI OR other citation details if **external detailed step-by-step protocols** are available.	Not Applicable	

Experimental study design and statistics	Information included in the manuscript?	In which section is the information available? (Reagents and Tools Table, Materials and Methods, Figures, Data Availability Section)
Include a statement about **sample size** estimate even if no statistical methods were used.	Yes	Materials and Methods
Were any steps taken to minimize the effects of subjective bias when allocating animals/samples to treatment (e.g. **randomization procedure**)? If yes, have they been described?	Yes	Materials and Methods
Include a statement about **blinding** even if no blinding was done.	Yes	Materials and Methods
Describe **inclusion/exclusion criteria** if samples or animals were excluded from the analysis. Were the criteria pre-established?<br><br>If sample or data points were omitted from analysis, report if this was due to attrition or intentional exclusion and provide justification.	Yes	Materials and Methods
For every figure, are **statistical tests** justified as appropriate? Do the data meet the assumptions of the tests (e.g., normal distribution)? Describe any methods used to assess it. Is there an estimate of variation within each group of data? Is the variance similar between the groups that are being statistically compared?	Yes	Materials and Methods

Sample definition and in-laboratory replication	Information included in the manuscript?	In which section is the information available? (Reagents and Tools Table, Materials and Methods, Figures, Data Availability Section)
In the figure legends: state number of times the experiment was **replicated** in laboratory.	Yes	In the Figure legends
In the figure legends: define whether data describe **technical or biological replicates**.	Yes	In the Figure legends

Ethics

Ethics	Information included in the manuscript?	In which section is the information available? (Reagents and Tools Table, Materials and Methods, Figures, Data Availability Section)
Studies involving **human participants**: State details of **authority granting ethics approval** (IRB or equivalent committee(s), provide reference number for approval.	Not Applicable	
Studies involving **human participants**: Include a statement confirming that **informed consent** was obtained from all subjects and that the experiments conformed to the principles set out in the WMA Declaration of Helsinki and the Department of Health and Human Services Belmont Report.	Not Applicable	
Studies involving **human participants:** For publication of **patient photos**, include a statement confirming that consent to publish was obtained.	Not Applicable	
Studies involving experimental **animals**: State details of **authority granting ethics approva**l (IRB or equivalent committee(s), provide reference number for approval. Include a statement of compliance with ethical regulations.	Not Applicable	
Studies involving **specimen and field samples:** State if relevant **permits** obtained, provide details of authority approving study; if none were required, explain why.	Not Applicable	

Dual Use Research of Concern (DURC)	Information included in the manuscript?	In which section is the information available? (Reagents and Tools Table, Materials and Methods, Figures, Data Availability Section)
Could your study fall under dual use research restrictions? Please check biosecurity documents and list of **select agents and toxins** (CDC): https://www.selectagents.gov/sat/list.htm	Not Applicable	
If you used a select agent, is the security level of the lab appropriate and reported in the manuscript?	Not Applicable	
If a study is subject to dual use research of concern regulations, is the name of the **authority granting approval and reference number** for the regulatory approval provided in the manuscript?	Not Applicable	

Reporting

The MDAR framework recommends adoption of discipline-specific guidelines, established and endorsed through community initiatives. Journals have their own policy about requiring specific guidelines and recommendations to complement MDAR.

Adherence to community standards	Information included in the manuscript?	In which section is the information available? (Reagents and Tools Table, Materials and Methods, Figures, Data Availability Section)
State if relevant guidelines or checklists (e.g., **ICMJE, MIBBI, ARRIVE, PRISMA**) have been followed or provided.	Yes	Materials and Methods
For **tumor marker prognostic studies,** we recommend that you follow the **REMARK** reporting guidelines (see link list at top right). See author guidelines, under 'Reporting Guidelines'. Please confirm you have followed these guidelines.	Not Applicable	
For **phase II and III randomized controlled trials**, please refer to the **CONSORT** flow diagram (see link list at top right) and submit the CONSORT checklist (see link list at top right) with your submission. See author guidelines, under 'Reporting Guidelines'. Please confirm you have submitted this list.	Not Applicable	

Data Availability

Data availability	Information included in the manuscript?	In which section is the information available? (Reagents and Tools Table, Materials and Methods, Figures, Data Availability Section)
Have **primary datasets** been deposited according to the journal's guidelines (see 'Data Deposition' section) and the respective accession numbers provided in the Data Availability Section?	Yes	Materials and Methods
Were **human clinical and genomic datasets** deposited in a public access-controlled repository in accordance to ethical obligations to the patients and to the applicable consent agreement?	Not Applicable	
Are **computational models** that are central and integral to a study available without restrictions in a machine-readable form? Were the relevant accession numbers or links provided?	Not Applicable	
If publicly available data were reused, provide the respective **data citations in the reference list**.	Not Applicable	
